# The Discovery of Highly Potent THP Derivatives as OCTN2 Inhibitors: From Structure-Based Virtual Screening to In Vivo Biological Activity

**DOI:** 10.3390/ijms21197431

**Published:** 2020-10-08

**Authors:** Francesca Di Cristo, Anna Calarco, Filomena Anna Digilio, Maria Stefania Sinicropi, Camillo Rosano, Umberto Galderisi, Mariarosa Anna Beatrice Melone, Carmela Saturnino, Gianfranco Peluso

**Affiliations:** 1 Elleva Pharma S.R.L., via Pietro Castellino 111, 80131 Naples, Italy; francesca.dicristo@ellevapharma.com; 2 Research Institute on Terrestrial Ecosystems (IRET)-CNR, Via Pietro Castellino 111, 80131 Naples, Italy; anna.calarco@cnr.it (A.C.); filomenaanna.digilio@cnr.it (F.A.D.); 3 Department of Pharmacy, Health and Nutritional Sciences, University of Calabria, Via P. Bucci, 87036 Arcavacata di Rende, Italy; s.sinicropi@unical.it; 4 Proteomics and Mass Spectrometry Unit, IRCCS Ospedale Policlinico San Martino, Largo R. Benzi 10, 16132 Genova, Italy; camillo.rosano@hsanmartino.it; 5 Department of Experimental Medicine, Biotechnology and Molecular Biology Section, Luigi Vanvitelli Campania University, Vico Luigi De Crecchio 1, 80138 Naples, Italy; umberto.galderisi@unicampania.it; 6 Department of Advanced Medical and Surgical Sciences, 2nd Division of Neurology, Center for Rare Diseases and InterUniversity Center for Research in Neurosciences, University of Campania “Luigi Vanvitelli”, via Sergio Pansini 5, 80131 Naples, Italy; marina.melone@unicampania.it; 7 Department of Science, University of Basilicata, Viale dell’Ateneo Lucano 10, 85100 Potenza, Italy

**Keywords:** carnitine system, carnitine/organic cation transporter (OCTN2), mitochondrial β-oxidation, Huntington’s disease (HD), meldonium

## Abstract

A mismatch between β-oxidation and the tricarboxylic acid cycle (TCA) cycle flux in mitochondria produces an accumulation of lipid metabolic intermediates, resulting in both blunted metabolic flexibility and decreased glucose utilization in the affected cells. The ability of the cell to switch to glucose as an energy substrate can be restored by reducing the reliance of the cell on fatty acid oxidation. The inhibition of the carnitine system, limiting the carnitine shuttle to the oxidation of lipids in the mitochondria, allows cells to develop a high plasticity to metabolic rewiring with a decrease in fatty acid oxidation and a parallel increase in glucose oxidation. We found that 3-(2,2,2-trimethylhydrazine)propionate (THP), which is able to reduce cellular carnitine levels by blocking both carnitine biosynthesis and the cell membrane carnitine/organic cation transporter (OCTN2), was reported to improve mitochondrial dysfunction in several diseases, such as Huntington’s disease (HD). Here, new THP-derived carnitine-lowering agents (TCL), characterized by a high affinity for the OCTN2 with a minimal effect on carnitine synthesis, were developed, and their biological activities were evaluated in both in vitro and in vivo HD models. Certain compounds showed promising biological activities: reducing protein aggregates in HD cells, ameliorating motility defects, and increasing the lifespan of HD *Drosophila melanogaster*.

## 1. Introduction

Carnitine is an important molecule that regulates mitochondrial long-chain fatty acid β-oxidation and enables the import of fatty acid intermediates from the cytosol to the mitochondria via the carnitine cycle [1]. A mismatch between β-oxidation and tricarboxylic acid cycle (TCA) flux, such as when a dysregulated carnitine cycle increases the mitochondrial fatty acid supply to a level that exceeds the demand and/or the enzymatic capacity, produces the accumulation of lipid metabolic intermediates, resulting in both blunted metabolic flexibility and decreased glucose utilization. The ability of the cell to switch to glucose as an energy substrate could be restored by reducing the reliance of the cell on fatty acid oxidation [2]. The inhibition of carnitine palmitoyltransferase 1 (CPT1), the key rate-limiting enzyme of the carnitine carrier system, decreases fatty acid oxidation with a parallel increase in glucose oxidation [3]. Although CPT1 inhibition limits the carnitine shuttle for the oxidation of long-chain lipids in the mitochondria, carnitine allows the system to work through the peroxisomal synthesis of short- and medium-chain acylcarnitine. These carnitine derivatives can enter into the mitochondrial matrix independently by CPT1, still leading to mitochondria oversupply and dysfunction. As carnitines are important for mitochondrial β-oxidation, a disturbance in plasma membrane carnitine transport and/or liver carnitine synthesis can significantly affect fatty acid oxidation.

Three-(2,2,2-trimethylhydrazine)propionate (THP; Meldonium), a structural analog of the carnitine precursor γ-butyrobetaine, reduces the cellular carnitine levels by suppressing endogenous carnitine biosynthesis via the inhibition of the γ-butyrobetaine hydroxylase (BBOX) enzyme [4] and by blocking the cell membrane carnitine/organic cation transporter (OCTN2). In THP-induced low-carnitine cells, THP diminishes β-oxidation by inhibiting fatty acid (FA) transport into the mitochondrial matrix [5] and modulates the energy metabolism by increasing glycolysis [6,7,8]. Recently, we demonstrated that THP was able to effectively restore the expression of peroxisome proliferator-activated receptor gamma coactivator 1-alpha (PGC-1α), a master regulator of mitochondria biogenesis, dynamics, and oxidative metabolism, in cell and animal models of Huntington’s disease (HD) [9,10]. 

These biological properties make THP a potentially useful treatment for diseases characterized by dysfunctional mitochondrial fatty acid oxidation and biogenesis, such as diabetes, angiocardiopathy, and some types of neuropathy. However, the decrease in hepatic carnitine levels, mainly due to BBOX inhibition, has been shown to give rise to fatty liver in animals [11,12]. A recent paper proposed a novel mechanism by which 5-aminovaleric acid betaine (5-AVAB), a bioactive compound present in diets rich in whole grains, can participate in the regulation of cellular energy metabolism. The study demonstrated that 5-AVAB, a structural analogue of THP, was able to reduce β-oxidation of fatty acids by decreasing the intracellular carnitine level via OCTN2 blocking, without affecting the biosynthesis of l-carnitine via BBOX [13].

Here, we developed and investigated new THP-derived carnitine-lowering agents (TCL) characterized by a high affinity for an OCTN2 carrier, with a minimal effect on BBOX activity. Understanding the impact of the selected TCL on carnitine uptake has allowed us to characterize cell metabolic adaptations induced by treatment with TCL. To test TCL, we used both cell and animal models of HD as research has demonstrated that the expression of mutated huntingtin (mHtt) induces mitochondrial dysfunction and metabolic remodeling [14,15,16].

A secondary aim of the study was to unravel the interplay between perturbations of the carnitine system and the rewiring of the metabolic cell phenotype to identify specific bioenergetic signatures to guide new approaches to therapeutic intervention. Our results revealed a shift toward the higher oxidation of glucose in TCL-treated HD cells, an effect that is directly associated with the inhibitory activity of the tested compound. Two compounds showed promising biological activity, decreasing mHtt aggregates in striatal cells expressing mHtt and ameliorating the motility defects of HD flies as well as increasing their lifespan.

## 2. Results

### 2.1. Molecular Docking Study of the Interactions between THP and OCTN2 and the Synthesis of THP Structurally-Related Compounds (TLC)

**Figure 1:**
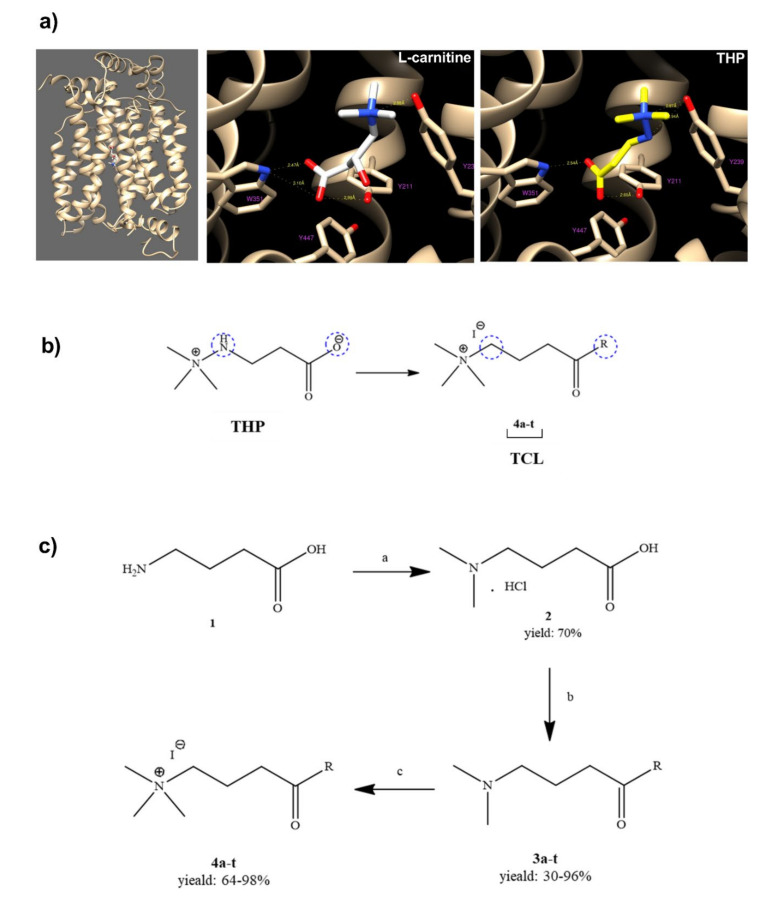
(**A**) Binding modes of l-carnitine (white sticks) THP (yellow sticks) resulting from docking simulations. Residues involved in protein‒ligand interactions are drawn as balls and sticks; the receptor hOCTN2 is reported in tan ribbons; (**B**) structural modifications carried out on THP; (**C**) synthesis of TCL **4a**–**t**. (a) HCOH 37%, HCOOH, 60 °C, 16 h; (b) for **3a**-**j** and **3l**-**t**: amine, DCC, HOBT, Et_3_N, CH_2_Cl_2_, or THF, RT, 24 h, for **3k**: 4-nitroaniline, PCl_3_, pyridine, 3 h, 40 °C; (c) CH_3_I, acetone, 18 h, RT. Abbreviations: 3-(2,2,2-trimethylhydrazine)propionate, THP; THP structurally related compounds, TCL; hydroxymethylene, HCOH; formic acid, HCOOH; *N*,*N*’- dicyclohexylcarbodiimide, DCC; hydroxybenzotriazole, HOBT; *N*,*N*-diethylethanamine, Et_3_N; dichloromethane, CH_2_Cl_2_; tetrahydrofurane, THF; room temperature, RT; phosphorus trichloride, iodomethane, PCl_3_.

To identify the interaction between THP (3-(2,2,2-trimethylhydrazine)propionate; Meldonium) and new synthetized THP structurally-related compounds (TCL) with OCTN2, a computational OCTN2 docking analysis was performed. As the crystal structure of the OCTN2 protein has not been resolved, a computational model of human OCTN2 (hOCTN2) was developed to create a rational ligand design. The carnitine binding site in the computational model of hOCTN2 was used to investigate the interactions between the transporter and THP or TCL. The docking simulations for the validated model of OCTN2 showed an interaction between Tyrosine 239 and the trimethylamine group of THP, as well as between the carboxylic acid group and both Tryptophan 351 and Tyrosine 211, similar to l-carnitine (Figure 1a). The docking simulations also highlight an interaction between the amine group (NH) of THP and Tyrosine 239. Based on molecular modeling, the structural modifications carried out on THP included the replacement of the functional groups more involved in the interactions with OCTN2 (Figure 1b). In particular, the –NH group was replaced with a –CH_2_ group, and the carboxyl group was transformed in highly functionalized aliphatic and aromatic amides (Figure 1c, compounds **4a**–**t**). The quaternary nitrogen, bearing the three methyl groups, was kept unaltered.

Before testing their activity on both BBOX and OCTN2, adequate in vitro monitoring for compound-related toxicity was carried out to exclude molecules affecting cell viability. All the compounds at a concentration of 50 µM were non-toxic and did not affect cell viability, with the exception of compound **4f** (Figure S1).

### 2.2. Evaluation of OCTN2-Mediated TCL Cellular Uptake and Their Carnitine Uptake Inhibition

**Figure 2:**
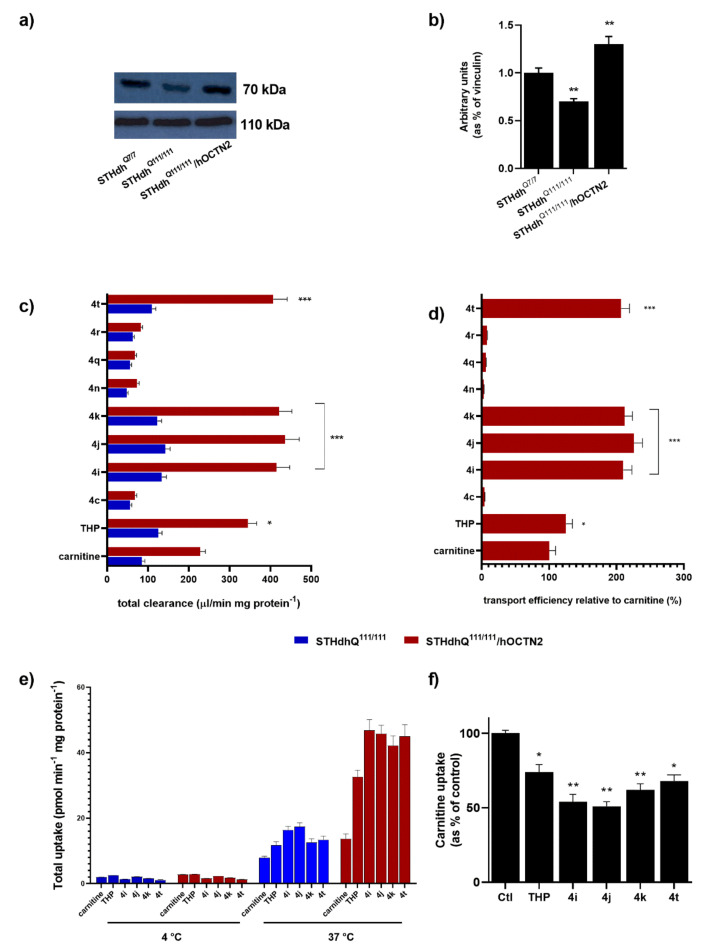
(**a**,**b**) Western blot analysis of hOCTN2 expression in STHdh^Q7/7^, STHdh^Q111/111^, and transfected STHdh^Q111/111^ (STHdh^Q111/111^/hOCTN2). ^§§^ *p* < 0.01 vs. STHdh^Q7/7^. (**c**) The total accumulation of carnitine (10 µM), THP, or TCL (50 μM) in STHdh^Q111/111^ cells with or without the heterologous expression of hOCTN2. (**d**) The efficiency of the transport of drugs by hOCTN2 in STHdh^Q111/111^ cells with the heterologous expression of hOCTN2. (**e**) Accumulation at 37 °C or at 4 °C of carnitine, THP, or TCL in STHdh^Q111/111^ cells with or without the heterologous expression of hOCTN2. (**f**) Carnitine uptake in OCTN2-transfected STHdh^Q111/111^ cells in the presence of 50 μM THP, and the newly synthesized compound. The bars represent the mean ± standard deviation (*n* = 3). Statistical significance: * *p* < 0.05, ** *p* < 0.01, *** *p* < 0.001 vs. CTL.

To reliably measure the transport activity, the stable hOCTN2 overexpression was obtained in STHdh^Q111/111^ (Figure 2a,b). An initial analysis of the OCTN2 expression on STHdh^Q111/111^ and the cognate STHdh cells expressing wild-type Htt (STHdh^Q7/7^ cells) demonstrated that striatal cells with Htt mutations showed a significant increase in OCTN2 compared with a wild-type counterpart (Figure 2a). The carnitine accumulation between STHdh^Q111/111^ with and without heterologous OCTN2 transporter expression depended on whether the cells were transfected with either hOCTN2 (100%) or the expression vector pCMV6-XL5 alone (18%). In the latter case, the carnitine uptake derived from the constitutive expression of OCTN2 in STHdh^Q111/111^. The carnitine uptake at pH 7.4, expressed as the cell-to-medium ratio, increased time-dependently in STHdh^Q111/111^ OCTN2-transfected cells for 2 min and attained steady-state uptake by 10 min, whereas the uptake by STHdh^Q111/111^ transfected with pCMV6-XL5 vector alone exhibited a significantly lower increase of uptake over 10 min. Thus, the uptake at 1 min was routinely used for the initial uptake rate measurement in the subsequent studies.

The total uptake of TCL into STHdh^Q111/111^ with and without the overexpression of hOCTN2 was then evaluated. The transporter-mediated uptake was calculated as the difference in the total uptake between cells with and without carrier overexpression. Figure 2c depicts the widely different levels of the accumulation of carnitine, THP, and several TCL in target cells. Transport efficiency relative to carnitine (100%) demonstrates that some tested TCL qualify as optimal substrates for hOCTN2, significantly better than THP itself (Figure 2d). The other TCL cannot be considered hOCTN2 substrates as their carrier-mediated uptake was mostly not significantly different from zero or was relative to carnitine. Data concerning TCL with low cell uptake are reported only for certain compounds. In addition, the intracellular accumulation of selected TCL, as well as carnitine and THP, was abolished almost entirely (<3% of control) at 4 °C (Figure 2e) or when extracellular Na^+^ was replaced with *N*-methyl-d-glucamine (NMG) at an equimolar concentration.

Finally, carnitine uptake by cultured STHdh^Q111/111^ was significantly inhibited by TCL substrates, including **4i**, **4j**, and **4k** (*p* < 0.01), and, to a lesser extent, by **4t** and THP (*p* < 0.05) (Figure 2f). Accordingly, **4i**, **4j**, **4k**, and **4t** were chosen for the subsequent experiments.

### 2.3. Evaluation of TCL’s Ability to Modulate Energetic Metabolism by a Targeted Drug Screen

**Figure 3:**
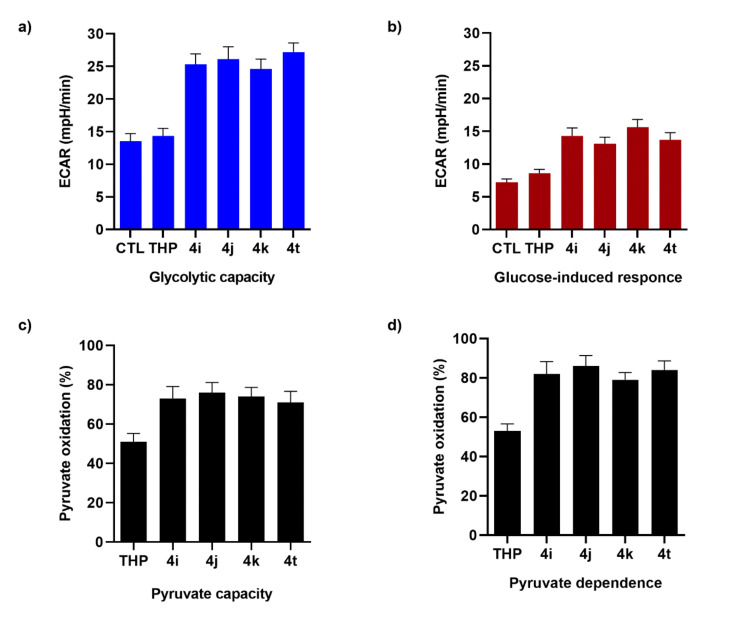
(**a**,**b**) STHdh^Q111/111^ treated with 50 μM of THP or selected TCL; (**c**,**d**) Bioenergetics studies of STHdh^Q111/111^ cultured in the presence of pyruvate as a specific energetic substrate. The percent capacity to use pyruvate for respiration as well as the percent dependence on pyruvate was calculated as described in the Materials and Methods Section 4.9. Each experiment was independently repeated at least three times, in separate 24-well plates with each treatment in 4–12 replicate wells.

To understand if selected TCL were able to reprogram STHdh^Q111/111^ to use glycolytic metabolites as an alternative fuel, the STHdh^Q111/111^ energetic metabolism was evaluated in the presence of TCL. STHdh^Q111/111^ treatment with TCL (**4i**, **4j**, **4k**, and **4t**) significantly increased both the glycolytic capacity and glucose-induced response, while THP treatment resulted in a non-significant increase in the extracellular acidification rate (ECAR) (Figure 3a). The oxygen consumption rate (OCR) of cultured STHdh^Q111/111^ demonstrated the cellular dependence on pyruvate to fuel mitochondrial respiration and the capacity for utilizing this substrate when the others (i.e., FA and glutamine) are blocked (Figure 3b). The reliance of untreated STHdh^Q111/111^ on pyruvate to fuel oxygen consumption was lower. The treatment with TCL had a very strong impact on the STHdh^Q111/111^ mitochondrial dependence on pyruvate importation into the mitochondria and the capacity for import when fatty acid oxidation and glutaminolysis were both inhibited (Figure 3c,d).

The dependence of the STHdh^Q111/111^ on pyruvate as a metabolic substrate doubled after TCL treatment (Figure 3d). We concluded that TCL treatment increased the ability of STHdh^Q111/111^ to use the cellular dependence on glucose and pyruvate to fuel metabolism, suggesting a recovery of the metabolic flexibility—seen as the ability of the cell to switch to glucose as an energy substrate when other energy substrates are quantitatively inadequate.

### 2.4. Evaluation of BBOX Inhibition by TCL

Endogenous carnitine synthesis plays an important role in carnitine physiological homeostasis. The rate-limiting step in this pathway involves the hydroxylation of BBOX to yield carnitine. Interaction studies between TCL and BBOX were conducted to establish if the molecular modifications carried out to THP interfere with BBOX activity. As reported in Table 1, TCL display negligible BBOX inhibition relative to THP due to the profound modifications made to its carboxyl group. In particular, the replacement of the carboxylate in the four selected TCL produced inactive compounds without an inhibitory effect on BBOX.

### 2.5. Computational Docking Studies of Interactions between OCTN2 and TCL Selected on the Basis of Their Inhibitory Effect on the Carnitine Uptake

**Figure 4:**
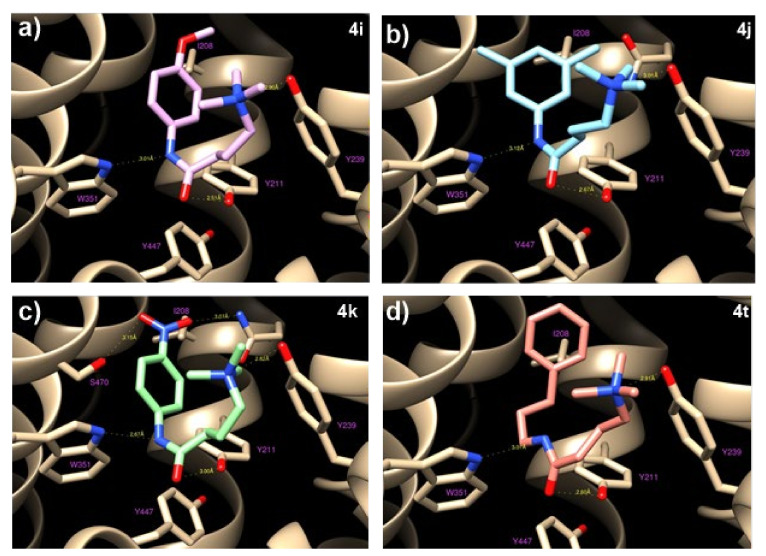
The binding modes of the different ligands tested, resulting from docking simulations. The receptor hOCTN2 is depicted by tan ribbons, and all the ligands are sticks. The residues involved in protein‒ligand interactions are drawn as shapes and sticks. (**a**,**b**) The binding mode for **4i** (pink sticks) and **4j** (light blue sticks); **4k** is in green in (**c**); (**d**) molecule **4t** is shown in orange.

To investigate the interactions between **4i**, **4j**, **4k**, and **4t** and OCTN2, as well as the structure−activity relationship, in silico docking studies were performed. Molecular modeling and docking simulations allowed us to identify the ligand’s binding site as a deep cavity encompassed by transmembrane helices TM1, TM4, TM5, TM7, TM10, and TM11 (Figure 4). The different hOCTN2 protein residues involved in ligand binding are reported in Table S3.

All of the selected compounds exhibited a higher binding affinity to OCTN2 compared to THP; this could be due to the replacing of COOH with longer fragments containing an aromatic ring, which allows for hydrophobic interactions with the residues Val 153, Ile 208, and Ser 470. This space remains empty in the case of THP, with a consequent lowering of the binding constants. On the other hand, the substitution of the amine with a ‒CH group did not seem to cause relevant variations because the hydrogen bond occurring between Tyr239 and the ‒NH group of THP is rather long (about 3 Å) and is, therefore, a weak link.

### 2.6. Cell Treatment with TCL Decreased mHtt Aggregation

Previous studies demonstrated that, in striatal cells expressing mHtt, compensatory shifts to generate energy from FAs may exacerbate mitochondrial dysfunction, leading to an increase of reactive oxygen species (ROS) production, which promotes, directly or indirectly, mHtt aggregation [17,18]. Emerging evidence has shown that mHtt aggregates induce cytotoxicity, which is closely related to neuronal death in HD [19,20], while the reduction in mHtt aggregates has been proven to rescue HD-related phenotypes [21]. Since the clearance of mHtt aggregates has become a promising strategy for HD therapy, we evaluated the effect of treatment with selected TCL on the level of mHtt aggregates in STHdhQ^111/111^.

**Figure 5:**
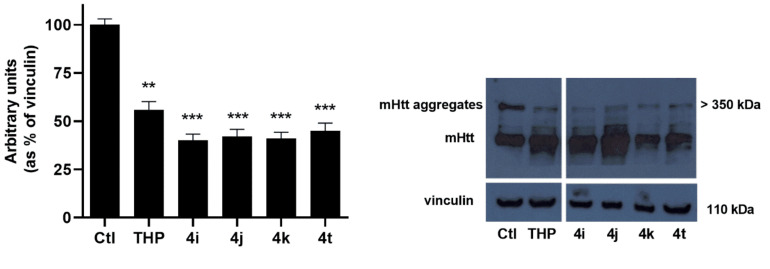
Representative western blot of mHtt aggregates in the STHdh^Q111/111^ cell line incubated for 72 h in the presence of of 50 μM **4i**, **4j**, **4k**, or **4t**. Densitometric quantification was performed on three different experiments, and the results are expressed as the mean of the values obtained (mean ± SD). ** *p* < 0.01 and *** *p* < 0.001 versus control.

Western blot analysis of STHdh^Q111/111^ incubated in the presence of the selected TCL for 72 h at a concentration of 50 μM demonstrated that cell treatment with the selected TCL significantly (*p* < 0.001) reduced mHtt aggregates (Figure 5). The results obtained are in agreement with the hypothesis that decreasing fatty acid oxidation (FAO) by modulating the carnitine system has beneficial effects on the expression of the pathological phenotype. In particular, the choice of OCTN2 as a druggable target is in line with our previous results, which demonstrated that the Na^+^-dependent carnitine transporter OCTN2 is overexpressed in STHdh^Q111/111^, thus contributing, through the import of carnitine into the cell, to FAO increase.

Certain studies offered more indirect evidence of the pathogenic role played by OCTN2 in HD. A recent paper reported that amino acids 14–22 and 447–454 of the OCTN2 sequence were involved in the interaction with caveolin-1, which directs this carrier to rafts [22]. The loss of caveolin-1 expression in a knock-in mouse model of Huntington’s disease suppresses the pathophysiology in vivo—a protective effect that might also be related to a decrease in the OCTN2 plasma membrane expression [23].

### 2.7. Selected TCL Rescue Neuronal Deficits in HD Flies

To confirm the efficacy of newly synthesized compounds in vivo, the selected compounds were tested in a Drosophila model of HD, with transgenic flies that express full-length human pathogenic cDNA, encoding a mutated HTT protein [9,24]. The expression of this construct in the transgenic fly recapitulated the majority of the pathological hallmarks of HD, including early death and locomotor dysfunction. In a recent previous work, Di Cristo et al. reported that THP significantly improved the lifespan and motor function in this HD fly and that the srl gene (DmPGC1-alpha) was involved in those effects [9]. In this work, we wanted to evaluate the lifespan and motor dysfunction of Q128HD-FL flies after treatment with the THP-derived molecules.

**Figure 6:**
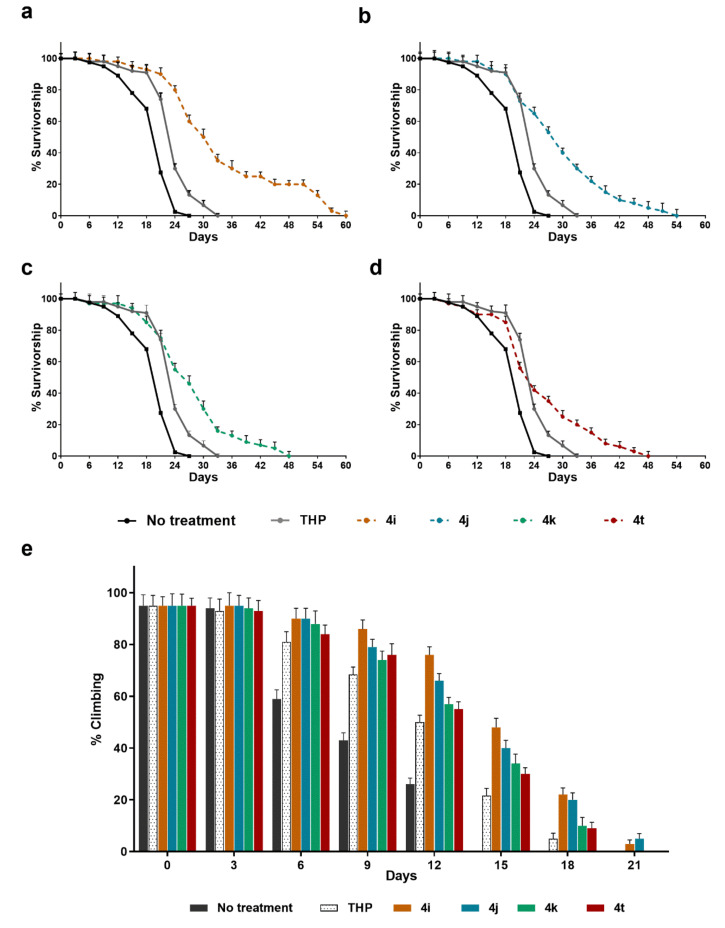
The effects of THP-derived molecules **4i**, **4j**, **4k**, and **4t** on fly lifespan and motor dysfunction. (**a**–**d**) Lifespan curves of transgenic UAS-Q128HD-FL/Elav-GAL4 flies fed with diets supplemented with the tested molecules. For each molecule, the comparison of age-dependent survival curves was done both with respect to the solvent alone and to THP. All treated groups had increased lifespans compared to both the control group and the THP group. (**a**) A longer extension in survival with respect to THP was observed in response to **4i**, (*p* < 0.001), with a 44.95% increase in the mean lifespan. (**b**) A dramatic comparable increase was obtained after treatment with **4j** (*p* < 0.001), with an increase in the mean lifespan of 25.3%. (**c**) A moderate but highly significant increase in survival was observed in response to **4k** (*p* < 0.001; mean lifespan +16.96%). (**d**) A minor increase in survival, although highly significant, was observed in response to **4t** (*p* < 0.01; mean lifespan +9.18%). *n* = 100 flies. (**e**) The climbing ability of UAS-Q128HD-FL/Elav-GAL4 transgenic flies fed with different media supplemented with one of four different derived-THP molecules, with THP, or with vehicle alone for control, was evaluated. All the tested THP-derived molecules improved the climbing ability of transgenic flies. The data are given as the mean ± SD. *n* = 60. *p* < 0.001 compared with THP for **4j**, **4i**, **4k**, and **4t**.

Transgenic flies were fed with six different foods: a control diet with a standard cornmeal Drosophila medium, and five media supplemented with THP or one of the four derived molecules. As shown in Figure 6, our results indicated that all the THP-derived molecules significantly extended the lifespans of the Q128HD-FL flies compared with THP-treated sibling flies. In particular, **4i** and **4j** dramatically extended the mean lifespan by as much as 44.95% and 20.6% respectively and the maximum lifespan by 88.13% and 42.37% compared to THP (Figure 6a,b). All four tested molecules significantly (*p* < 0.001) improved the survival rate of Q128HD-FL flies with respect to THP. After an initial overlap period of 21 days, the respective curves diverged as a function of the increased survival of the different fly groups. The survival curves of all four groups declined slowly compared to the curve of the THP group, at between 18 and 57 days, without a quick drop.

In addition, the influence of these molecules on the locomotion of the flies was assessed, as a parameter of improved health. Due to damaged motor neuronal function, the HD flies showed progressive movement disorders that can be evaluated by the climbing test, which takes advantage of the natural tendency of flies to climb a vial wall. Our results indicate that all four THP-derived molecules significantly ameliorated the climbing ability of transgenic flies (Figure 6e). For all the molecules, motor dysfunction appeared later, with a higher percentage of flies that achieved the target (10 s) over time with respect to the THP-treated flies (*p* < 0.001). In terms of lifespan, better results were obtained after treatment with **4i** and **4j** as shown in Figure 6e.

The beneficial effects of metabolic reprogramming in HD flies, with a shift toward glucose as an energy source, has already been reported. The overexpression of glucose-6-phosphate dehydrogenase, the major enzyme for the pentose phosphate pathway (PPP) [25], or overexpression of a glucose transporter [26], extended the lifespan in flies expressing 93 glutamine repeats.

Several other active compounds able to modulate the carnitine system have been proposed for the treatment of pathologies characterized by mitochondrial dysfunction. Here, we focused on one of the most important components of the carnitine shuttle, the OCTN2 carnitine carrier, as a possible druggable target. Selected TCL were able to reduce the negative impact of mHtt expression in cell and animal models of Huntington’s disease. This could occur through the inhibitory effect of TCL on OCTN2-mediated carnitine uptake, which negatively influenced the contribution of fatty acids to the overall ATP production, thus, reducing FA mitochondrial overloading and, in turn, breaking the futile FAO‒ROS cycle, a harbinger of pathological events. This suggestion is corroborated by studies on other THP structural analogs, such as 5-aminovaleric acid betaine (5-AVAB), which reduced the oxygen consumption caused by the β-oxidation of fatty acids in cultured cardiomyocytes by inhibiting OCTN2.

THP, the parent structure of TCL, was recently added to a list of prohibited drugs by the World Anti-Doping Agency, although there is not a full scientific consensus on its efficacy as a performance-enhancing agent. In addition, some authors have questioned the safety of THP, arguing that a reduction in β-oxidation might be detrimental to those tissues that rely on a fatty-acid-derived energy metabolism. These questions need to be addressed in future studies, although the loss of BBOX inhibition from TCL should reduce the side effects in the liver.

## 3. Discussion

Metabolic flexibility is an essential attribute for the maintenance of cell homeostasis and relates to the plasticity of the cell to adapt its metabolism to nutrients present in the microenvironment to oxygen availability and to hormonal input. Generally speaking, the cell may utilize both glucose and FA to meet its high energy demands [27], but normally high glycolytic flux and FA oxidation do not simultaneously occur (the glucose fatty-acid cycle or Randle cycle). Flux through these metabolic pathways is influenced by different factors, such that, at different physiologic conditions, glucose and FA are used in different proportions and neither are over-accumulated. The dysregulation of the Randle cycle may contribute to the dysregulation of mitochondria and induce cytotoxic effects [28].

In particular, full oxidation of FA generates certain metabolites that inhibit glycolysis. In the case of mitochondrial dysfunction by a dysregulated FA catabolism, high rates of fatty acid oxidation increase the mitochondrial acetyl-CoA/free CoA and NADH/NAD^+^ ratio, that, in turn, activates pyruvate dehydrogenase (PDH) kinase causing the phosphorylation and inhibition of PDH. PDH inhibition blocks the conversion of pyruvate to acetyl-CoA, decreases the influx of acetyl-CoA from glycolysis into the TCA cycle, and thereby reduces the cell metabolic flexibility with overreliance of the FA substrate. In these pathological conditions, inhibitors of FA β-oxidation appear to improve mitochondrial dysfunction and cell survival.

Recently, the inhibition of OCTN2-mediated carnitine entry into the cells by THP was shown to improve the control of diseases characterized by mitochondrial dysfunction, including neurodegenerative disease [29].

OCTN2 is the plasma membrane transporter involved in the absorption, distribution, and excretion of carnitine, and is also responsible for the traffic (absorption and secretion) of carnitine derivatives and analogs [30]. THP was first reported as a competitive inhibitor of OCNT2 [17] and, only later, was demonstrated to be an excellent substrate of OCTN2. The efficiency of THP transport by OCTN2 is even higher than that of carnitine. Cellular accumulation, transporter affinity, and sodium dependence indicate that THP mimics carnitine, although it lacks the hydroxyl group and contains a hydrazinium residue [13]. In addition, while inhibiting the absorption of carnitine, THP simultaneously competes with γ-butyrobetaine (γ-BB) for γ-butyrobetaine hydroxylase (BBOX) binding to inhibit carnitine endogenous synthesis, thus, exacerbating the carnitine deficiency and promoting the formation of liver steatosis [18].

This study reports the design and synthesis of new THP-related compounds, characterized by an increased ability to bind OCTN2 with a negligible effect on BBOX activity by in silico docking simulations. Other studies reported the synthesis of THP-related compounds by studying the crystal structures of BBOX complexed with γ-BB or THP steatosis. In this case, the target was to select improved inhibitors of BBOX, even if some molecules also displayed OCTN2 inhibition activity [18]. To confirm the efficacy of the new synthesized compounds both in vitro and vivo, in our study, the THP-related compounds were tested in STHdh^Q111/111^ cells and in a Drosophila model of HD.

The choice of these models derives from the fact that we demonstrated, in a previous study, the benefit of THP treatment on reducing both mHtt aggregation and the severity of the disease. To date, the experimental results provided evidence that, in the HD brain, glycolytic striatal cells reprogrammed their fuel use and switched to FA oxidation as an alternative energy source. These metabolic changes were linked to mitochondrial dysfunction and neuronal cell death [31]. Accordingly, while an increase in fatty acid oxidation (FAO) maintains the bioenergetic capacity of striatal mitochondria, escalating β-oxidation creates a futile FAO‒ROS (reactive oxygen species) cycle in which the energy-producing FAO pathway becomes the primary source of ROS-dependent damage. Intriguingly, the increased ROS production leads to increased mHtt aggregation [32].

The results reported demonstrated that only cells treated with selected THP-related compounds showed a significant recovery of the glycolytic capacity accompanied by a concomitant decrease of FAO addiction. The rewiring of the metabolism by THP-related compounds was associated with a significant reduction of mHtt aggregation in STHdh^Q111/111^ cells. This last effect is of interest as convincing evidence has demonstrated that decreasing mHtt by many different approaches can rescue HD-associated phenotypes [33].

Consistent with the cell experiments, we observed an improvement of motor function-related deficits as well as the lifespan of Q128HD-FL flies after the treatment with selected THP-related compounds. The beneficial effects of metabolic reprogramming in HD flies, with a shift toward glucose as an energy source, was previously reported. The overexpression of glucose-6-phosphate dehydrogenase, the major enzyme for the pentose phosphate pathway (PPP) [25], or overexpression of a glucose transporter [26], was shown to extend the lifespan in flies expressing 93 glutamine repeats.

## 4. Materials and Methods

### 4.1. Reagents and Equipment

All reactions were performed using commercially available compounds without further purification. All reagents used were of analytical grade and were purchased from Sigma-Aldrich (Milan, Italy). The column chromatographic purification of products was carried out using silica gel 60 (70–230 mesh, Merck, Milan, Italy). The NMR spectra were recorded on Bruker (Karlsruhe, Germany) DRX 400, 300, 250 spectrometers (400 MHz, 300 MHz, 250 MHz, 1H; 100 MHz, 75 MHz, 62.5 MHz ^13^C). The spectra were referenced to residual CHCl3 (7.26 ppm, 1H, 77.23 ppm, ^13^C). The coupling constants J are reported in Hz. Yields are given for isolated products that showed one spot on a thin-layer chromatography plate and no impurities detectable in the NMR spectrum. Mass spectral analyses were carried out using an electrospray spectrometer Waters 4 micro quadrupole. l-(methyl-3*H*)Carnitine hydrochloride (65 Ci/mmol) was purchased from Moravec Biochemicals (Brea, CA, USA).

Dulbecco’s modified Eagle’s medium (DMEM), fetal bovine serum (FBS), streptomycin–penicillin, l-glutamine, and sodium pyruvate were purchased from EuroClone (Milan, Italy). The Cell Proliferation Kit I (3-(4,5-dimethylthiazol-2-yl)-2,5-diphenyl tetrazolium bromide (MTT)) was obtained from Sigma-Aldrich. The following western blot antibodies were used in this study: anti-OCTN2 (1:1000, TA327136, Origene, Milan, Italy), anti-polyglutamine-expansion (1:2000, MAB1574, Merck, Milan, Italy), anti-vinculin (1:25000, AB129002, Abcam, Milan, Italy), and anti-mouse and anti-rabbit peroxidase-conjugated secondary antibodies (Biorad, Rome, Italy). Other reagents, including those for cell culture, membrane preparation, and transport experiments, were obtained from Sigma-Aldrich, Wako Pure Chemical Industries (Osaka, Japan), and Life Technologies (Milan, Italy).

### 4.2. General Method for the Preparation of THP-Derived Carnitine-Lowering Agents (TCL)

4.2.1. 4-(Dimethylamino) Butanoic Acid Hydrochloride **2**

A mixture of γ-amino butyric acid 1 (1 g, 9.71 mmol), 1.94 mL of formaldehyde (59 mmol, six 6 equivalents), and 2.52 mL of formic acid (67 mmol, 8.9 equivalents) was refluxed at 60 °C for 16 h. After cooling the solution, 1.2 mL of concentrated hydrochloric acid was added, and the water was removed under reduced pressure. Crystallization with acetonitrile produced a white solid. Yield: 71%. m.p. 97–98 °C (acetonitrile). MS (ESI) *m*/*z*: 132.2 (M + H) + ^1^H NMR (D_2_O, 300 MHz) δ: 3.05–2.99 (m, 2H), 2.73 (s, 6H), 2.34 (t, 2H, J = 7.2), 1.90–1.79 (m, 2H). ^13^C NMR (D_2_O, 75 MHz) δ: 177.3, 58.5, 45.9, 33.8, 23.2.

#### 4.2.2. General Procedure for the Synthesis of Amides **3a–t**

To a solution of **2** (50 mg, 0.31 mmol) in dichloromethane or tetrahydrofuran (1 mL) under magnetic stirring, dicyclohexylcarbodiimide (0.31 mmol), 1-hydroxybenzotriazole (0.31 mmol), triethylamine (0.45 mmol), and suitable amine (0.25 mmol) were added. The reaction was stirred overnight at room temperature. The residue, rinsed with dichloromethane, was extracted with 1 N HCl; the acidic phase was basified with NaOH 1N and extracted three times with dichloromethane. Step-by-step synthetic process can be found in Section 1 in the Supplementary Materials.

#### 4.2.3. General Procedure for the Synthesis of Ammonium Salts **4a–t**

A solution of compounds **3a**–**t** (1 equiv.) in acetone (1 mL) was reacted with two equivalents of iodomethane overnight. Crystallization with diethyl ether produced the desired products with good yields. Step-by-step synthetic process can be found in Section 1 in the Supplementary Materials.

### 4.3. Molecular Modeling and Docking Simulations

A computational model of hOCTN2 was built using the web server Phyre2 [34], and its three-dimensional structure was used as a target for the subsequent docking simulations. To evaluate the binding modes and calculated affinities between the newly synthesized THP analogues and hOCTN2, we used a “blind-docking approach” (no a priori information about the binding site was provided to the system). Molecular docking studies were performed using the programs Autodock and ADT [35,36] using a searching box encompassing the whole internal cavity of the protein (i.e., the whole protein, excluding the surface, was embedded in the lipidic membrane). All the ligand atomic structures were built and their three-dimensional coordinates’ energy minimized using the program MarvinSketch (ChemAxon Ltd., Budapest, Hungary). The docking experiment consisted of 100 cycles of Lamarckian Genetic Algorithm. The resulting poses were ranked according to their docking energy values and further clustered on the basis of a root-mean-square deviation (RMSD) cutoff value of 2.0 Å. Such a procedure allowed us to define the binding modes and calculate the binding energy with precision. All the figures were drawn with the program Chimera [37].

### 4.4. Cell Cultures

Conditionally immortalized wild-type STHdh^Q7/7^ and mutant STHdh^Q111/111^ striatal neuronal cell lines (kind gifts of Elena Cattaneo, Milan, Italy) were derived from striata of wild-type Hdh^Q7/7^ and homozygous Hdh^Q111/111^ littermate embryos [38]. The cells expressed endogenous levels of normal and mutant full-length huntingtin protein with seven (wild-type STHdh^Q7/7^) and 111 (STHdh^Q111/111^) glutamines, respectively. Striatal cells were grown as described by Di Cristo et al. [9].

### 4.5. Cell Viability

The cell viability was assessed using the (3-(4,5-Dimethylthiazol-2-yl)-2,5-Diphenyltetrazolium Bromide) MTT Cell Proliferation Assay. Briefly, cells (5 × 10^3^/well) were seeded in 96-well plates and treated with increasing concentrations of THP or structurally related compounds (50 μM) in the presence of DMEM containing 10% FBS. The absorbance was read at 550 nm on a Cytation 3 Imaging Reader (ASHI, BioTek Instruments, Milan, Italy), after 24, 48, and 72 h. The cell survival in the presence of **4a**–**t** compounds was compared to nontreated cells.

### 4.6. Western Blotting

Protein lysates from striatal cell lines were used for sodium dodecyl sulfate-polyacrylamide gel electrophoresis (SDS-PAGE) and western blot analyses. SDS-PAGE and western blots were carried out according to standard procedures and in triplicate. Relative expressions, normalized to the housekeeping protein, were quantified densitometrically using ImageJ Software (ImageJ; NIH).

### 4.7. Uptake Studies

To obtain STHdh^Q111/111^ cells (STHdh^Q111/111^) overexpressing OCTN2, STHdh^Q111/111^ were transfected with plasmid DNA (solute carrier family 22 member 5 (SLC22A5) (NM_003060) Human Untagged Clone) containing hOCTN2 cDNA (pCMV6-XL5/hOCTN2, Origene), and then selected following a procedure already described [39]. STHdh^Q111/111^/hOCTN2 was used for uptake experiments at a confluence of at least 70%. The uptake was measured at 37 °C and 4 °C in the presence of an uptake buffer (125 mM NaCl, 25 mM HEPES pH 7.4, 5.6 mM glucose, 4.8 mM KCl, 1.2 mM KH_2_PO_4_, 1.2 mM CaCl_2_, and 1.2 mM MgSO_4_) containing 50 μM unlabeled THP, TCL, or (*N*-methyl-3*H*)-carnitine (at 0.1 µM). A sodium-free transport medium was also used to assess the Na^+^-dependent uptake of the tested compounds. A sodium-free transport medium was prepared by replacing 125 mM NaCl in the transport medium with 125 mM *N*-methyl-d-glucamine (NMG) Cl; this was used to assess the cellular uptake in the absence of Na^+^.

To evaluate the inhibitory effect of THP or TCL on the L-carnitine cell uptake, the STHdh^Q111/111^/hOCTN2 monolayers were washed with uptake buffer and pre-incubated with THP or TCL (10 or 100 µM) for 15 min at 37 °C. The carnitine uptake was initiated by the simultaneous addition of unlabeled carnitine (10 µM) and (*N*-methyl-3*H*)-carnitine (0.1 µM; 12 kBq/mL) for a 60 min incubation at 37 °C. After STHdh^Q111/111^/hOCTN2 incubation with the different mixtures, the culture medium or uptake buffer was removed and the cells were washed with ice-cold PBS. Labeled carnitine was determined after cell lysis with 0.1% *v*/*v* Triton X-100 in 5 mM Tris-HCl, pH 7.4, by liquid scintillation counting. For THP and TCL determination, the cells were solubilized with 4 mM HClO4 or methanol and stored at −20° C. After centrifugation (1 min, 16,000× *g*, 20 °C) of the thawed lysates, the supernatant was analyzed by ultra-performance liquid chromatography-tandem mass spectrometry (UPLC/MS/MS) to determine the l-carnitine amount (given as the ratio to the control). The protein concentration was determined by a BCA protein assay.

### 4.8. Evaluation of BBOX Inhibition by TCL

The assay procedure for the evaluation of BBOX activity, which has been described in detail previously [40,41,42], was performed, measuring the formation of carnitine from gamma-butyrobetaine (GBB). Human recombinant BBOX was used as an enzyme source. The reaction mixture (20 mM potassium phosphate, pH 7.0, 20 mM potassium chloride, 3 mM 2-oxoglutarate, 0.25 mM ferrous ammonium sulfate, 10 mM sodium ascorbate, 0.16 mg of catalase, 200 μM GBB, and 0.6 μg of BBOX) was preincubated for 15 min in the presence or absence (control) of the tested inhibitor (100 or 1000 μM). The reaction was initiated by adding GBB, and, to ensure the linear rate range, the mixture was incubated at 37 °C for 30 min (human BBOX). The reaction was stopped with 0.8 mL of ice-cold acetonitrile:methanol (1:3 *v*/*v*). The determination of carnitine was performed by ultraperformance liquid chromatography−tandem mass spectrometry (UPLC/MS/MS) in a positive-ion electrospray.

### 4.9. Metabolic Fuel Flux Assays

Seahorse Glycolysis Stress and Mito Fuel Flex tests were carried out on a Seahorse XF24 Analyzer (Agilent, Milan, Italy). All assays were performed following the manufacturer’s protocols at 24 h post-incubation with medium alone, THP, or selected TCL. For the Glycolysis Stress Test, untreated or treated STHdhQ111/111 were washed and equilibrated in the glycolysis stress test medium with glutamine (2 mM) for 1 h. The experimental groups were also supplemented with the respective selected compounds during the assay. The basal extracellular acidification rate (ECAR) was determined first. Then, 10 mM glucose was injected, followed by 1 μM oligomycin (to inhibit oxidative phosphorylation), with the glycolytic capacity calculated by subtracting the basal ECAR from the maximal rate following both injections. The glucose-induced response was calculated by subtracting the basal ECAR from the maximal ECAR after glucose injection. The resulting decrease in ECAR after the final injection of 2-deoxy-glucose, a glycolysis inhibitor, confirmed that the ECAR produced in the experiment was due to glycolysis.

The oxygen consumption rate (OCR) in cultured STHdh^Q111/111^ was measured with Mito Fuel Flex Tests on a Seahorse XF24 Analyzer following the manufacturer’s protocols. Briefly, the percent capacity to use pyruvate for respiration was calculated by dividing the OCR from pyruvate alone by the OCR with pyruvate, FA, and glutamine import inhibited. The Seahorse Mito Fuel Flex Test Kits contains all three pathway inhibitors (UK5099, BPTES, and Etomoxir) for glucose, glutamine, and fatty acids, respectively. The percent dependence on pyruvate was estimated from the difference in basal OCR and pyruvate-blocked OCR divided by the basal OCR less the OCR after all FA and glutamine importation was blocked with sequential injections of 2 μM UK5099, followed by a combined 4 μM etomoxir and 3 μM bis-2(5-phenylacetamido-1,2,4-thiadiazol-2-yl)ethylsulfide (BPTES), respectively.

### 4.10. Drosophila Stocks

Flies were reared on standard corn meal agar with a 12 h on–off light cycle at 25 °C. Fly stocks used in the current study were obtained from the Bloomington Stock Center (Bloomington, IN, USA): 33808 w*; P{UAS-HTT.128Q.FL}f27b-8765 w;P{GAL4-elav.L}2-1521 w[*]; P{w[ + mC] = UAS-GFP.S65T}Myo31DF[T2].

### 4.11. THP Treatment and Crosses

THP-derived molecules (**4i**, **4j**, **4k**, and **4t**) were added into the surface of the assay fly food (AF: 2% agar, 10% powdered yeast, 10% sucrose, and 0.1% Nipagin), and left under gentle agitation for 3 h at room temperature until dried. This food was used for growing experimental flies, while the controls were reared in AF supplemented with the vehicle alone. Water was supplemented in equal amounts in all the food conditions. Based on the bipartite expression system upstream activator sequence (UAS)-GAL4 [43] the expression of the UAS-HTT.128Q. FL gene was obtained by crossing females carrying the pan-neural driver elav-Gal4 to males from the UAS HTT128QFL strain. The parental strains elav/+ as well as UAS HTT128QFL and P{w[ + mC] = UAS-GFP.S65T}T2/P{GAL4-elav.L}2) were used as controls. For all assays, only one sex was used in the study.

### 4.12. Lifespan Assay

Lifespan assay was carried out as previously described [44]. Briefly, newly emerged adult flies with the desired genotype (P{UASHTT.128Q.FL}f27b/P{GAL4-elav.L}2) were collected under cold-induced anesthesia, sorted by sex, grouped into five cohorts of 20 individuals in vials containing 3.5 mL of AF supplemented or not with the different molecules, and reared at 28 °C. Subsequently, flies were transferred to new vials, with fresh food once every three days. The lifespan was measured by recording the number of dead flies at each transfer, until no living flies remained in the vials. Each lifespan measurement used 100 flies, and this was repeated in three independent experiments per treatment. The values obtained were used to calculate the mean lifespan (the mean survival days of all flies for each group) and maximum lifespan (the maximum number of days needed to reach 90% mortality).

### 4.13. Negative Geotaxis Assay

Briefly, 20 sex-matched flies were placed in a graduated empty plastic vial (18 Å, 2.5 cm), and allowed to recover for at least 60 min. Negative geotaxis was measured by recording the number of flies that climbed above the 10 cm mark within 10 s after a tap-down of the flies to the bottom of the vial. This assay was repeated for the same group two times, allowing for a 1 min rest period between each trial. The number of flies per group that passed the 10 cm mark was recorded as a percentage of total flies. Each trial was performed three times at each time point, and the data were expressed as an average of the replicates (*n* = 300).

### 4.14. Statistical Analyses

All quantitative data were presented as the mean ± SD, and the statistical significance was evaluated using one-way ANOVA analysis, followed by a post hoc Bonferroni’s test for multiple comparisons to determine any statistical differences between groups. Each experiment was performed at least three times. Asterisks were used to indicate a significant difference from the controls (* *p* < 0.05, ** *p* < 0.01, and *** *p* < 0.001). All the data were analyzed with the GraphPad Prism v. 5.01 statistical software package (GraphPad, La Jolla, CA, USA).

## 5. Conclusions

There is increasing experimental evidence that mutant Htt (mHtt) aggregates play an important role in the pathogenesis of HD in human and model organisms [45,46]. Indeed, a key pathological feature of HD is the aberrant accumulation of mHtt protein into high molecular weight complexes. An increase of mHtt aggregates in mouse brain extracts concomitantly with the appearance of symptoms, suggests that this quantitatively tracks disease progression. Mechanistic studies with an inducible Drosophila model of HD indicated a correlation between the appearance of mHtt aggregates in adult neurons and the reduced survival of HD flies, again supporting the hypothesis that mHtt aggregate formation is a disease-relevant process [47].

Other studies indicated that mHtt is a druggable target as antagonizing mHtt protein aggregations in HD cell and animal models can inhibit severe downstream phenotypic changes [48]. Many further steps are needed to develop selected compounds for clinical use. Once a high-efficacy compound has been identified by optimizing the structure of THP, additional screenings must be carried out. For example, the compounds must be tested for their penetration of the blood–brain barrier, the pharmacokinetic/pharmacodynamic properties, and the safety profiles for further drug discovery purposes. Finally, we intend to continue our study on the selected molecules and to assess their ability to affect PGC1α levels and activity.

## Figures and Tables

**Table 1:** The inhibitory effect of TCL on γ-butyrobetaine hydroxylase (BBOX).

Substance	Human BBOX IC_50_ (µM)
THP	61 ± 10
**4i**	89 ±12
**4j**	97 ± 17
**4k**	78 ± 16
**4t**	76 ± 13

## Supplementary Materials

Supplementary materials can be found at https://www.mdpi.com/1422-0067/21/19/7431/s1. Section 1 describes step-by-step synthetic process of TCL. Table S1. Amines used for the preparation of **4a–t.** Table S2. Ammonium salts 4a-t. Table S3. The different hOCTN2 protein residues involved in ligand binding. Figure S1. Cell viability of STHdh^Q7/7^ and STHdh^Q111/111^ cell lines incubated for 72 h in presence of 50 μM of compounds. Figure S2. Kinetic profile of ECAR in STHdh^Q111/111^ cells treated with THP or selected TCL.
