# Supplementary material for: The Discovery of Highly Potent THP Derivatives as OCTN2 Inhibitors: From Structure-Based Virtual Screening to In Vivo Biological Activity
Source: Int J Mol Sci. 2020 Oct 8;21(19):7431. doi: 10.3390/ijms21197431 (PMC7583931; doi:10.3390/ijms21197431)
Supplement: Supplementary file 1 [file ijms-21-07431-s001.pdf]

International Journal of *Molecular Sciences*

Supplementary Materials

The Discovery of Highly Potent THP Derivatives as OCTN2 Inhibitors: From Structure-Based Virtual Screening to in Vivo Biological Activity

Francesca Di Cristo [1], Anna Calarco [2,†], Filomena Anna Digilio [2], Maria Stefania Sinicropi [3], Camillo Rosano [4], Umberto Galderisi [5], Mariarosa Anna Beatrice Melone [6], Carmela Saturnino [7,*] and Gianfranco Peluso [2,*]

1. Elleva Pharma S.R.L. via Pietro Castellino, 111, 80131 Naples, Italy; francesca.dicristo@ellevapharma.com
2. Research Institute on Terrestrial Ecosystems (IRET)-CNR, Via Pietro Castellino 111, 80131 Naples, Italy; anna.calarco@cnr.it (A.C.); filomenaanna.digilio@cnr.it (F.A.D.)
3. Department of Pharmacy, Health and Nutritional Sciences, University of Calabria, Via P. Bucci, 87036 Arcavacata di Rende, Italy; s.sinicropi@unical.it
4. Proteomics and Mass Spectrometry Unit. IRCCS Ospedale Policlinico San Martino, Largo R. Benzi 10, 16132 Genova, Italy; camillo.rosano@hsanmartino.it
5. Department of Experimental Medicine, Biotechnology and Molecular Biology Section, Luigi Vanvitelli Campania University, Vico Luigi De Crecchio, 1, 80138 Naples, Italy; umberto.galderisi@unicampania.it
6. Department of Advanced Medical and Surgical Sciences, 2nd Division of Neurology, Center for Rare Diseases and InterUniversity Center for Research in Neurosciences, University of Campania "Luigi Vanvitelli", via Sergio Pansini, 5, 80131 Naples, Italy; marina.melone@unicampania.it
7. Department of Science, University of Basilicata, Viale dell'Ateneo Lucano 10, 85100 Potenza, Italy

* Correspondence: carmela.saturnino@unibas.it (C.S.); gianfranco.peluso@cnr.it (G.P.)

† This author contributed to this work as senior author.

Section 1

Synthesis of THP structurally related compounds

4. -(dimethylamino)-*N*-propylbutanamide 3a

Waxysolid. Yield:75%. MS (ESI) *m/z*: 172.16(M + H)$^+$. ^{1}H NMR ($CDCl_3$, 400 MHz) δ: 6.52 (bs, 1H), 3.17 (q, 2H, *J*= 6.6 Hz), 2.30-2.23 (m, 2H), 2.20 (m + s, 8H), 1.76 (t, 2H, *J*= 6.8 Hz), 1.49 (q, 2H, *J*= 7.2 Hz), 0.90 (t, 3H, *J*= 7.3 Hz).^{13}C NMR($CDCl_3$, 100 MHz) δ: 172.7, 58.1, 45.9, 42.6, 34.2, 24.1, 23.2, 11.2.

4. -(dimethylamino)-*N*-butylbutanamide 3b

Waxysolid. Yield: 74%. MS (ESI) *m/z***:** 186.17(M + H)$^+$. ^{1}H NMR ($CDCl_3$, 300 MHz) δ: 6.50 (bs, 1H), 3.21 (q, 2H, *J*= 7.0 Hz), 2.47 (t, 2H, *J*= 6.8 Hz), 2.34 (s, 6H), 2.26 (t, 2H, *J*= 7.0 Hz), 1.86–1.81 (m, 2H), 1.48–1.43 (m, 4H), 0.90 (t, 3H, *J*= 7.2 Hz). ^{13}C NMR($CDCl_3$, 75 MHz) δ**:** 172.7, 58.1, 45.9, 40.1, 34.2, 32.3, 24.1, 19.9, 13.8.

4. -(dimethylamino)-*N*-(prop-2-ynyl)butanamide 3c

Waxysolid. Yield:72%. MS (ESI) *m/z*: 168.13(M + H)$^+$. ^{1}H NMR ($CDCl_3$, 300 MHz) δ:7.67 (bs, 1H), 4.01-3.99 (m, 2H), 2.32 (q, 4H, *J*= 7.0 Hz), 2.21 (s, 6H + 1H), 1.61 (q, 2H, *J*= 6.5 Hz). ^{13}C NMR($CDCl_3$, 75 MHz) δ: 173.0, 78.1, 70.9, 58.1, 45.9, 33.3, 30.0, 24.1.

4. -(dimethylamino)-*N*-(3-methoxypropyl)butanamide 3d

Waxysolid. Yield:74%. MS (ESI) *m/z*: 202.17(M + H)$^+$. ^{1}H NMR ($CDCl_3$, 400 MHz) δ: 6.64 (bs, 1H), 3.42 (t, 2H, *J*= 5.4 Hz), 3.22 (s, 3H), 2.41 (t, 2H, *J*= 6.6 Hz), 2.18 (s, 10H), 1.78-1.73 (m, 4H). ^{13}C NMR ($CDCl_3$, 100 MHz) δ: 172.7, 71.7, 59.3, 58.1, 45.9, 36.7, 29.6, 24.1.

4. -(dimethylamino)-*N*-(hexadecyl)butanamide 3e

Waxysolid. Yield:31%. MS (ESI) *m/z*: 354.5 (M + H)$^+$**.** ^{1}H NMR ($CDCl_3$, 400 MHz) δ:6.44 (bs, 1H), 3.20 (t, 2H, *J*= 6.22 Hz), 2.29 (t, 2H, *J*=6.6 Hz), 2.23 (m+s, 8H), 1.77 (t, 2H, *J*= 6.8 Hz), 1.46 (s, 2H), 1.25 (s, 26H), 0.86 (d, 3H, *J*= 7.0 Hz).^{13}C NMR ($CDCl_3$, 100 MHz) δ: 172.7, 58.1, 45.9, 40.4, 34.2, 31.9, 30.1, 29.7, 29.4, 26.8, 24.1, 22.8, 14.1.

N-cyclohexyl-4-(dimethylamino)butanamide 3f

Waxy solid. Yield:32%. MS (ESI) *m/z*: 212.3(M + H)$^+$. ^{1}H NMR ($CDCl_3$, 300 MHz) δ:6.49 (bs, 1H), 3.72 (d, 1H, *J*= 4.0 Hz), 2.26 (d, 1H, J= 2.9 Hz), 2.25 (s, 6H), 1.89–1.58 (m, 8H), 1.54–1.06 (m, 7H).^{13}C NMR ($CDCl_3$, 75 MHz) δ: 172.4, 58.1, 47.4, 45.9, 33.8, 34.5, 28.0, 24.1, 22.9.

4. -(dimethylamino)-1-(piperidin-1-yl)butan-1-one 3g

Waxy solid. Yield: 81%. MS (ESI) *m/z***:** 198.2 (M + H)$^{+.}$ ^{1}H NMR ($CDCl_3$, 400 MHz) δ: 3.68 (t, 2H, *J*= 5.4 Hz), 3.62 (t, 2H, *J*= 7.3 Hz), 2.43–2.36 (m, 4H), 2.19 (s, 6H), 1.78 (t, 2H, *J*= 7.6 Hz), 1.60 (d, 2H, *J*= 5.2 Hz), 1.53–1.50 (m, 4H). ^{13}C NMR ($CDCl_3$, 100 MHz) δ: 147.7, 58.1, 45.9, 44.8, 32.0, 25.6, 25.4, 24.4.

4. -(dimethylamino)-*N*-(phenyl)butanammide 3h

Compound was purified by chromatography on silica gel in a gradient, using as eluent a mixture of $CHCl_3/CH_3OH$ (90:10) to elute the impurities and a mixture of $CH_2Cl_2/CH_3OH/Et_3N$ (90:10:0.2) to elute the compound as an oil .Yield: 35%. MS (ESI) *m/z*: 207.3 (M+H)$^+$. ^{1}H NMR ($CDCl_3$, 300 MHz) δ: 9.77 (bs, 1H), 7.55 (d, 2H, *J*= 7.7 Hz), 7.31 (t, 2H, *J*= 5.8 Hz), 7.26 (bs, 1H), 2.55-2.53 (m, 4H), 2.37 (s, 6H), 1.93-1.89 (m, 2H). ^{13}C NMR ($CDCl_3$, 75 MHz) δ: 173.0, 138.5, 129.0, 124.4, 121.6, 58.1, 45.9, 33.8, 24.1.

4-(dimethylamino)-*N*-(4-methoxyphenyl)butanamide3i

Compound was purified by chromatography on silica gel in a gradient, using as eluent a mixture of $CHCl_3/CH_3OH$ 90:10 to elute the impurities and a mixture of $CH_2Cl_2/CH_3OH/Et_3N$ (90:10:0.5) to elute the compound as an oil. Yield: 75%. MS (ESI) *m/z*: 236.3 $(M + H)^+$. ^1H NMR ($CDCl_3$, 300 MHz) δ: 9.56 (bs, 1H), 7.45 (d, 2H, *J*= 8.9 Hz), 6.82 (d, 2H, *J*= 8.9 Hz), 3.75 (s, 3H), 2.57-2.49 (m, 4H), 2.38 (s, 6H), 1.92-1.87 (m, 2H). ^{13}C NMR ($CDCl_3$, 75 MHz) δ: 173.0, 156.3, 130.8, 122.6, 114.5, 58.1, 55.9, 45.9, 33.8, 24.1.

4. -(dimethylamino)-*N*-(3,4-dimethylphenyl)butanamide 3j

Compound was purified by chromatography on silica gel in a gradient, using as eluent a mixture of $CHCl_3/CH_3OH$ 90:10 to elute the impurities and a mixture of $CH_2Cl_2/CH_3OH/Et_3N$ 90:10:0.5 to elute the compound. Oil. Yield: 67% MS (ESI) *m/z*: 235.3 $(M + H)^+$. ^1H NMR ($CDCl_3$, 300 MHz) δ: 9.41 (bs, 1H), 7.34 (s, 1H), 7.21 (d, 1H, *J*= 7.8 Hz), 7.03 (d, 1H, *J*= 7.8 Hz), 2.47-2.38 (m, 4H), 2.27 (s, 6H), 2.17 (s, 6H), 1.86–1.83 (m, 2H). ^{13}C NMR ($CDCl_3$, 75 MHz) δ: 173.0, 137.1, 135.4, 132.5, 129.2, 118.5, 58.1, 45.9, 33.8, 24.1, 17.8.

4-(dimethylamino)-*N*-(4-nitrophenyl)butanamide3k

To a solution of 4-nitroaniline (82.9 mg, 0.6 mmol, 2 eq) in pyridine (1 mL), phosphorous trichloride (26.2 μL, 0.3 mmol, 1 equivalent) at room temperature was added and stirring was kept for 1 hour. Then 50 mg of **2** (0.31 mmol) were put into the reaction mixture and temperature brought at 40 °C for three hours. Pyridine was removed under vacuum and the residue rinsed with dichloromethane was extracted with 1N HCl. The acidic phase was basified with NaOH 1N and extracted for three times with dichloromethane. Product was obtained as an oil. Yield: 72%. MS (ESI) *m/z*: 252.3 $(M + H)^+$. ^1H NMR ($CDCl_3$, 400 MHz) δ: 8.16 (d, 2H, *J*= 9.0 Hz), 7.66 (d, 2H, *J*= 9.0 Hz), 2.56 (t, 2H, *J*= 6.2 Hz), 2.49 (t, 2H, *J*= 5.7 Hz), 2.34 (s, 6H), 1.86 (t, 2H, *J*= 5.9 Hz). ^{13}C NMR ($CDCl_3$, 100 MHz) δ: 173.0, 144.6, 144.0, 122.5, 121.3, 58.1, 45.9, 33.8, 24.1.

4. -(dimethylamino)-*N*-(benzyl)butanamide 3l

Compound was purified by chromatography on silica gel in a gradient, using as eluent a mixture of $CHCl_2/CH_3OH$ 90:10 to elute the impurities and a mixture of $CH_2Cl_2/CH_3OH/Et_3N$ (90:10:0.2) to elute the compound. Waxy solid. Yield: 87%. MS (ESI) *m/z*: 219.2 $(M + H)^+$. ^1H NMR ($CDCl_3$, 300 MHz) δ: 7.29 (q 5H, *J*= 6.4 Hz), 4.41 (d, 2H, *J*= 5.5 Hz), 2.34-2.17 (m, 4H), 2.08 (s, 6H), 1.18-1.75 (m, 2H). ^{13}C NMR ($CDCl_3$, 75 MHz) δ: 173.0, 141.7, 128.6, 127.0, 126.8, 58.1, 45.9, 44.1, 34.2, 24.1.

4-(dimethylamino)-*N*-(4-chlorobenzyl)butanamide3m

Oil. Yield: 42%. MS (ESI) *m/z*: 255.8 $(M + H)^+$. ^1H NMR ($CDCl_3$, 300 MHz) δ: 7.34-7.19 (m, 5H), 4.37 (d, 2H, *J*= 5.6 Hz), 2.34–2.26 (m, 4H), 2.11 (s, 6H), 1.80-1.76 (m, 2H). ^{13}C NMR ($CDCl_3$, 75 MHz) δ: 173.0, 139.8, 132.3, 128.7, 128.4, 58.1, 45.9, 34.2, 24.1.

4-(dimethylamino)-*N*-(4-methoxybenzyl)butanamide3n

Oil. Yield: 49% MS (ESI) *m/z*: 251.3 $(M + H)^+$. ^1H-NMR ($CDCl_3$, 250 MHz) δ: 7.19 (d, 2H, *J*= 8.6 Hz), 6.84 (d, 2H, *J*= 8.6 Hz), 4.32 (d, 2H, *J*= 5.4 Hz), 3.78 (s, 3H), 2.29–2.24 (m, 4H), 2.09 (s, 6H), 1.79–1.73 (m, 3H). ^{13}C NMR ($CDCl_3$, 60 MHz) δ: 173.0, 158.7, 134.0, 128.0, 114.1, 58.1, 55.9, 45.9, 44.1, 34.2, 24.1.

4. -(dimethylamino)-*N*-(4-nitrobenzyl)butanamide 3o

Product was purified by chromatography on silica gel in a gradient, using as eluent a mixture of $CHCl_3/CH_3OH$ (90:10) to elute the impurities and a mixture of $CH_2Cl_2/CH_3OH/Et_3N$ (90:10:0.2) to elute the compound as an oil. Yield: 43%. MS (ESI) *m/z*: 266.1 $(M+H)^+$. ^1H NMR ($CDCl_3$, 300 MHz) δ: 8.15 (d, 2H, *J*= 8.5 Hz), 7.77 (bs, 1H), 7.43 (d, 2H, *J*= 8.4 Hz), 4.49 (d, 2H, *J*= 5.8 Hz), 2.41–2.36 (m, 4H),

2.21 (s, 6H), 1.86–1.81 (m, 2H). ^{13}C NMR ($CDCl_3$, 75 MHz) δ: 173.0, 147.8, 146.4, 127.9, 120.9, 58.1, 45.9, 44.1, 34.2, 24.1.

(*RS*)-4-(dimethylamino)-*N*-(1-phenylethyl)butanamide 3p

Product was purified by chromatography on silica gel in a gradient, using as eluent a mixture of $CHCl_3/CH_3OH$ 90:10 to elute the impurities and a mixture of $CH_2Cl_2/CH_3OH/Et_3N$ 90:10:0.5 to elute the compound.Waxy solid. Yield: 30%. MS (ESI) *m/z*: 236.3 (M + H)$^+$. ^{1}H NMR ($CDCl_3$, 300 MHz) δ: 7.43 (bs, 1H), 7.33–7.22 (m, 5H), 5.09–5.04 (m, 1H), 2.67 (t, 2H, *J*= 6.7 Hz), 2.49 (s, 6H), 2.37–2.34 (m, 2H), 1.95 (t, 2H, *J*= 6.8 Hz), 1.47 (d, 3H, *J*= 6.5 Hz). ^{13}C NMR ($CDCl_3$, 75 MHz) δ: 172.7, 143.5, 128.6, 127.0, 126.8, 58.1, 49.5, 45.9, 34.5, 24.1.

4-(dimethylamino)-*N*-(3,5-dimethoxybenzyl)butanamide3q

Product was purified by chromatography on silica gel in a gradient, using as eluent a mixture of $CHCl_3/CH_3OH$ (90:10) to elute the impurities and a mixture of $CH_2Cl_2/CH_3OH/Et_3N$ (90:10:0.5) to elute the compound as an oil. Yield: 81%. MS (ESI) *m/z*: 281.2 (M + H)$^+$. ^{1}H NMR ($CDCl_3$, 300 MHz) δ: 7.21 (bs, 1H), 6.46 (s, 2H), 6.36 (s, 1H), 4.37 (d, 2H, *J*= 5.6 Hz), 3.80 (s, 6H), 2.59 (t, 2H, *J*= 6.7 Hz), 2.47–2.43 (m, 2H), 2.40 (s, 6H), 1.98–1.96 (m, 2H). ^{13}C NMR ($CDCl_3$, 75 MHz) δ: 173.0, 161.5, 143.7, 103.3, 98.4, 58.1, 55.9, 45.9, 44.7, 34.2, 24.1.

.4-(dimethylamino)-*N*-(4-metoxyphenylethyl)butanamide3r

Waxy solid. Yield: 92%. MS (ESI) *m/z*: 265.4 (M + H)$^+$. ^{1}H NMR ($CDCl_3$, 250 MHz) δ: 7.09 (d, 2H, *J*= 7.9 Hz), 6.82 (d, 2H, *J*= 7.9 Hz), 6.42 (bs, 1H), 3.77 (s, 3H), 3.48–3.43 (m, 2H), 2.73 (t, 2H, *J*= 7.0 Hz), 2.23–2.17 (m, 4H), 2.13 (s, 6H), 1.75–1.69 (m, 2H). ^{13}C NMR ($CDCl_3$, 60 MHz) δ: 172.7, 157.9, 131.8, 128.8, 114.2, 58.1, 55.9, 45.9, 40.7, 35.7, 34.2, 24.1.

4. -(dimethylamino)-N-(4-nitrofeniletil)butanamide 3s

Product was purified by chromatography on silica gel in a gradient, using as eluent a mixture of $CHCl_3/CH_3OH$ (90:10) to elute the impurities and a mixture of $CH_2Cl_2/CH_3OH/Et_3N$ (90:10:0.2) to elute the compoundas an oil. Yield: 46%. MS (ESI) *m/z*: 280.3 (M + H)$^+$. ^{1}H NMR ($CDCl_3$, 300 MHz) δ: 8.16 (d, 2H, *J*= 8.5 Hz), 7.37 (d, 2H, *J*= 8.3 Hz), 3.51 (t, 2H, *J*= 6.7 Hz), 3.00–2.91 (m, 3H), 2.56 (t, 1H, *J*= 7.2 Hz), 2.26–2.18 (m, 4H), 2.15 (s, 6H), 1.74 (t, 1H, *J*= 6.9 Hz).^{13}C NMR ($CDCl_3$, 75 MHz) δ: 172.7, 145.6, 128.7, 121.0, 58.1, 45.9, 40.7, 35.7, 34.2, 24.1.

4. -(dimethylamino)-*N*-(3-phenylpropyl)butanamide 3t

Product was purified by chromatography on silica gel in a gradient, using as eluent a mixture of $CHCl_3/CH_3OH$ 90:10 to elute the impurities and a mixture of $CH_2Cl_2/CH_3OH/Et_3N$ 90:10:0.2 to elute the compound as an oil. Yield: 49%. MS (ESI) *m/z*: 247.2 (M + H)$^+$. ^{1}H NMR ($CDCl_3$, 300 MHz) δ: 7.18 (q, 5H, *J*=6.4 Hz), 6.95 (bs, 1H), 9.62 (t, 2H, *J*= 5.9 Hz), 2.60 (t, 2H, *J*= 7.6 Hz), 2.28 (t, 2H, *J*= 6.8 Hz), 2.17 (t, 8H, *J*= 7.4 Hz), 1.81–1.72 (m, 4H).^{13}C NMR ($CDCl_3$, 75 MHz) δ: 172.7, 138.1, 128.9, 128.2, 126.1, 58.1, 45.9, 40.0, 34.2, 33.1, 29.1, 24.1.

4. -(propylamino)-*N,N,N*-trimethyl-4-oxobutan-1-aminium iodide 4a

White solid. Yield: 79%. m.p.:129–130 °C. MS (ESI) *m/z*: 187.2 (M + H)$^+$. ^{1}H NMR ($CDCl_3$, 300 MHz) δ:6.61 (bs, 1H), 3.70–3.68 (m, 2H), 3.32 (s, 9H), 2.54 (t, 2H, *J*= 7.05 Hz), 2.22 (t, 2H, *J*= 7.2 Hz), 1.72 (s, 4H), 0.94 (t, 3H, *J*= 7.3 Hz). ^{13}C NMR ($CDCl_3$, 75 MHz) δ: 172.7, 66.0, 54.7, 42.6, 35.0, 23.2, 22.8, 11.2.

4. -(butylamino)-*N,N,N*-trimethyl-4-oxobutan-1-aminium iodide 4b

Yellow powder. Yield:37%. m.p.:119–120 °C. MS (ESI) *m/z*: 201.2 (M + H)$^+$. ^{1}H NMR ($CDCl_3$, 300 MHz) δ:6.58 (bs, 1H), 3.33 (s, 9H), 3.20 (q, 2H, *J*= 6.9 Hz), 2.71 (s, 2H), 2.32 (t, 2H, *J*= 6.6 Hz), 1.77 (t,

2H, *J*= 6.8 Hz), 1.44 (t, 2H, *J*= 7.1 Hz), 1.33 (t, 2H, *J*= 7.6 Hz), 0.89 (t, 3H, *J*= 72 Hz). ^{13}C NMR ($CDCl_3$, 75 MHz) δ: 172.7, 66.0, 54.7, 40.1, 35.0, 32.3, 22.8, 19.9, 13.8.

4. -(prop-2-ynylamino)-*N,N,N*-trimethyl-4-oxobutan-1- aminiumiodide 4c

Pale yellowpowder. Yield: 36%. m.p.:145 °C (dec.). MS (ESI) *m*/*z*: 183.15 (M + H)$^+$. ^{1}H NMR ($CDCl_3$, 300 MHz) δ:7.62 (bs, 1H), 4.01-3.98 (m, 2H), 3.31 (s, 9H), 2.32 (q, 4H, *J*= 7.0 Hz), 2.21 (m,1H), 1.61 (q, 2H, *J*= 6.5 Hz). ^{13}C NMR ($CDCl_3$, 300 MHz) δ: 173.0, 78.1, 70.9, 66.0, 54.7, 34.1, 30.0, 22.8.

4. -(methoxypropylamino)-*N,N,N*-trimethyl-4-oxobutan-1- aminiumiodide 4d

White powder. Yield: 78%. m.p.:137–138 °C. MS (ESI) *m*/*z*: 217.3 (M + H)$^+$. ^{1}H NMR ($CDCl_3$, 300 MHz) δ:6.69 (bs, 1H), 3.83-3.46 (m, 2H), 3.47 (t, 2H, *J*= 5.8 Hz), 3.33 (s, 3H), 3.38-3.35 (m + s, 11H), 2.44 (d, 2H, *J*= 6.6 Hz), 2.17–2.14 (m, 2H), 1.79 (t, 2H, *J*= 6.1 Hz)..^{13}C NMR ($CDCl_3$, 75 MHz) δ: 172.7, 71.7, 66.0, 59.3, 54.7, 36.7, 35.0, 29.6, 22.8.

4. -(hexadecylamino)-*N,N,N*-trimethyl-4-oxobutan-1- aminiumiodide 4e

Tan powder. Yield: 71%. M.p.139–140 °C. MS (ESI) *m*/*z*: 369.4 (M + H)$^+$.^{1}H NMR ($CDCl_3$, 300 MHz) δ:6.23 (bs, 1H), 3.65–3.63 (m, 2H), 3.27 (s, 9H), 2.53–2.52 (m, 2H), 2.22-2.20 (m, 2H), 1.51 (d, 10H, *J*= 8.5 Hz), 1.27 (d, 20H, *J*= 7.5 Hz), 0.92 (t, 3H, *J*= 9.4 Hz). ^{13}C NMR ($CDCl_3$, 75 MHz) δ: 172.7, 66.0, 54.7, 40.4, 35.0, 31.9, 30.1, 29.7, 26.8, 22.8, 14.1.

4. -(cyclohexylamino)-*N,N,N*-trimethyl-4-oxobutan-1- aminiumiodide 4f

Tan powder. Yield: 68%. m.p.:124–125 °C. MS (ESI) *m*/*z*: 227.2(M + H)$^+$. ^{1}H NMR ($CDCl_3$, 300 MHz) δ:6.64 (d, 1H, *J*= 7.9 Hz), 3.75–3.66 (m, 2H), 3.39 (s, 9H), 2.43 (t, 2H, *J*= 6.8 Hz), 2.12 (t, 2H, *J*= 8.0 Hz), 1.86-1.56 (m, 5H), 1.32–1.19 (m, 6H).^{13}C NMR($CDCl_3$, 75 MHz) δ: 172.4, 66.0, 54.7, 47.4, 35.3, 33.8, 28.0, 22.9, 22.8.

4. -(trimethylammonio)-1-(piperidin-1-yl)butan-1-one iodide 4g

Pale yellow powder. Yield: 84%. m.p.:132–134 °C. MS (ESI) *m*/*z*: 213.2(M+H)$^+$. ^{1}H NMR ($CDCl_3$, 300 MHz) δ:3.80–3.74 (m, 2H), 3.52–3.51(m, 2H), 3.39 (s, 9H), 2.54 (t, 2H, *J*= 6.1 Hz), 2.16–2.08 (m, 2H), 1.65–1.53 (m, 8H). ^{13}C NMR ($CDCl_3$, 75 MHz) δ: 174.7, 66.0, 54.7, 44.8, 32.8, 25.6, 25.4, 23.1

***N,N,N*-trimethyl-4-oxo-4-(phenylamine)butan-1- aminiumiodide 4h**

Pale oil.Yield: 69%. MS (ESI) *m*/*z*: 222.3 (M + H)$^+$. ^{1}H NMR (CD_3OD, 300 MHz) δ: 7.57 (d, 2H, *J*= 8.0 Hz), 7.30 (t, 2H, *J*= 7.9 Hz), 7.09 (s, 1H), 3.48–3.43 (m, 2H), 3.18 (s, 9H), 2.55 (t, 2H, *J*= 6.9 Hz), 2.15 (t, 2H, *J*= 8.1 Hz). ^{13}C NMR (CD_3OD, 75 MHz) δ: 173.0, 138.5, 129.0, 124.4, 121.6, 66.0, 54.7, 34.6, 22.8.

4. -[(4-methoxyphenyl)amino-*N,N,N*-trimethyl-4-oxobutan-1-aminium iodide 4i

White powder. Yield: 83%. m.p: 189–190 °C. MS (ESI) *m*/*z*: 252.2 (M + H)$^+$. ^{1}H NMR ($CDCl_3$, 300 MHz) δ: 7.44 (d, 2H, *J*= 8.8 Hz), 6.80 (d, 2H, *J*= 8.9 Hz), 3.19 (s, 3H), 3.61-3.55 (m, 2H), 3.15 (d, 9H, *J*= 7.1 Hz), 2.54 (t, 2H, *J*= 6.8 Hz), 2.09 (t, 2H, *J*= 8.4 Hz). ^{13}C NMR ($CDCl_3$, 75 MHz) δ: 173.0, 156.3, 130.8, 122.6, 114.5, 66.0, 55.9, 54.7, 34.6, 22.8.

4. -[(3,4-dimethylphenyl)amino]-*N,N,N*-trimethyl-4-oxobutan-1-aminiumiodide 4j

Yellow waxy gel. Yield: 82% MS (ESI) *m*/*z*: 250.4 (M + H)$^+$.^{1}H NMR ($CDCl_3$, 300 MHz) δ: 7.37 (d, 2H, *J*= 7.8 Hz), 7.02 (d, 1H, *J*= 8.4 Hz), 3.77-3.74 (m, 2H), 3.31 (s, 9H), 2.69 (t, 2H, *J*= 6.8 Hz), 2.19 (d, 6H, *J*= 3.3 Hz), 1.71 (s, 2H). ^{13}C NMR ($CDCl_3$, 75 MHz) δ: 173.0, 137.1, 135.4, 132.5, 129.2, 121.2, 117.5, 66.0, 54.7, 34.6, 22.8, 17.8.

4. -[(4-nitrophenyl)amino]-*N,N,N*-trimethyl-4-oxobutan-1-aminium iodide4k

White solid.Yield: 72%. m.p.: 244–245 °C (dec.). MS (ESI) *m*/*z*: 265.2 $(M + H)^+$. ^1H NMR ($CDCl_3$, 300 MHz) δ: 8.14 (d, 2H, *J*= 7.0 Hz), 7.77 (d, 2H, *J*= 6.9 Hz), 3.53-3.51 (m, 2H), 3.30 (s, 3H), 3.16 (s, 6H), 2.61 (m, 2H), 2.12 (m, 2H).^{13}C NMR ($CDCl_3$, 75 MHz) δ: 173.0, 144.6, 122.5, 121.3, 66.0, 54.7, 34.6, 22.8.

***N,N,N*-trimethyl-4-oxo-4-(benzylamino)butan-1-aminiumiodide 4l**

White solid. Yield: 98%. m.p.: 187–188 °C (dec.). MS (ESI) *m*/*z*: 234.2 $(M + H)^+$. ^1H NMR (DMSO-d_6, 400 MHz) δ: 7.32-7.22 (m, 5H), 4.25 (s, 2H), 3.26-3.23 (m, 2H), 3.12 (s, 9H), 2.20 (t, 2H, *J*= 7.2 Hz), 1.93–1.88 (m, 2H). ^{13}C NMR (DMSO-d_6, 100 MHz) δ: 173.0, 141.7, 128.6, 127.0, 126.8, 66.0, 54.7, 44.1, 35.0, 22.8.

4. -[(4-chlorobenzyl)amino]-*N,N,N*-trimethyl-4-oxobutan-1-aminiumiodide 4m

White solid. Yield: 98%. m.p:108–109 °C. MS (ESI) *m*/*z*: 269.8 $(M + H)^+$. ^1H NMR ($CDCl_3$, 300 MHz) δ: 7.28 (m, 4H), 7.11 (bs, 1H), 4.36 (d, 2H, *J*= 5.9 Hz), 3.84–3.78 (m, 2H), 3.30 (s, 9H), 2.54 (t, 2H, *J*= 6.6 Hz), 2.18–2.13 (m, 2H). ^{13}C NMR ($CDCl_3$, 75 MHz) δ: 173.0, 139.8, 132.3, 128.7, 128.4, 66.0, 54.7, 44.1, 35.0, 22.8.

4. -[(4-methoxybenzyl)amino]-*N,N,N*-trimethyl-4-oxobutan-1-aminiumiodide 4n

White solid. Yield: 89%. m.p.: 110–111 °C. MS (ESI) *m*/*z*: 265.5 $(M + H)^+$. ^1H NMR ($CDCl_3$, 300 MHz) δ: 7.20 (d, 2H, *J*= 7.3 Hz), 6.87 (d, 2H, *J*= 7.1 Hz), 4.29 (s, 2H), 3.76 (s, 3H), 3.36 (s, 2H), 3.13 (s, 9H), 2.33 (t, 2H, *J*= 6.7 Hz), 2.09-2.04 (m, 2H).^{13}C NMR ($CDCl_3$, 75 MHz) δ: 173.0, 158.7, 134.0, 128.0, 114.1, 66.0, 55.9, 54.7, 44.1, 35.0, 22.8.

***N,N,N*-trimethyl-4-[(4-nitrobenzyl)amino]-4-oxobutan-1-aminium iodide4o**

Orange gel. Yield: 89%. MS (ESI) *m*/*z*: 279.4 $(M + H)^+$. ^1H NMR ($CDCl_3$, 300 MHz) δ: 8.18 (d, 2H, *J*= 8.5 Hz), 7.54 (d, 2H, *J*= 8.4 Hz), 4.49 (s, 2H), 3.47–3.42 (m, 2H), 3.19 (s, 9H), 2.45 (t, 2H, *J*= 7.1 Hz), 2.17–2.00 (m, 2H).^{13}C NMR ($CDCl_3$, 75 MHz) δ: 173.0, 147.8, 146.4, 127.9, 120.9, 66.0, 54.7, 44.1, 35.0, 22.8.

(*RS*)-*N,N,N*-trimethyl-4-oxo-4-[(1-phenylethyl)amino]butan-1-aminium iodide 4p

White solid. Yield: 73%. m.p.: 153–154 °C. MS (ESI) *m*/*z*: 249.4 $(M + H)^+$. ^1H NMR ($CDCl_3$, 300 MHz) δ: 7.36–7.26 (m, 5H), 7.22 (bs, 1H), 5.06-4.99 (m, 1H), 3.41-3.34 (m, 2H), 3.16 (s, 9H), 2.46–2.38 (m, 2H), 2.12–2.00 (m, 2H), 1.48 (d, 3H, *J*= 7.0 Hz). ^{13}C NMR ($CDCl_3$, 75 MHz) δ: 172.7, 143.5, 128.6, 127.0, 126.8, 66.0, 54.7, 49.5, 35.3, 22.8, 21.6.

4. -[(3,5-dimethoxybenzyl)amino]-*N,N,N*-trimethyl-4-oxobutan-1-aminium iodide 4q

White solid.Yield: 98%. M.p.: 86–87 °C. MS (ESI) *m*/*z*: 295.4 $(M + H)^+$. ^1H NMR ($CDCl_3$, 250 MHz) δ: 8.06 (bs, 1H), 6.98 (s, 2H), 6.81 (s, 1H), 4.80 (d, 2H, *J*= 5.6 Hz), 4.26 (s, 6H), 4.15–4.12 (m, 2H), 3.79 (s, 9H), 2.97 (t, 2H, *J*= 6.7 Hz), 2.61 (m, 2H). ^{13}C NMR ($CDCl_3$, 75 MHz) δ: 173.0, 161.5, 143.7, 103.3, 98.4, 66.0, 55.9, 54.7, 44.7, 22.8.

4. -[(4-methoxyfenilethyl)amino]-*N,N,N*-trimethyl-4-ossobutan-1-aminium iodide 4r

Waxy solid. Yield: 84%. MS (ESI) *m*/*z*: 279.4 $(M + H)^+$. ^1H NMR ($CDCl_3$, 300 MHz) δ: 7.15 (d, 2H, *J*= 6.6 Hz), 6.84 (d, 2H, *J*= 6.6 Hz), 6.46 (bs, 1H), 3.77 (s, 3H), 3.69 (t, 2H, *J*= 8.2 Hz), 3.50–3.46 (m, 2H), 3.32 (s, 9H), 2.79 (t, 2H, *J*= 7.0 Hz), 2.42 (t, 2H, *J*= 6.3 Hz), 2.12–2.07 (m, 2H). ^{13}C NMR ($CDCl_3$, 75 MHz) δ: 172.7, 157.9, 131.8, 128.8, 114.2, 66.0, 55.9, 54.7, 40.7, 35.7, 35.0, 22.8.

***N,N,N*-trimethyl-4-[(4-nitrophenylethyl)amino]-4-ossobutan-1-aminium iodide 4s**

Yellow oil. Yield: 92%. MS (ESI) *m/z*: 293.4 (M + H)$^+$. ^{1}H NMR ($CDCl_3$, 300 MHz) δ: 8.16 (d, 2H, *J*= 8.4 Hz), 8.10 (bs, 1H), 7.48 (d, 2H, *J*= 8.4 Hz), 3.50-3.30 (m, 4H), 3.16 (s, 9H), 2.95 (t, 2H, *J*= 6.9 Hz), 2.30 (t, 2H, *J*= 6.9 Hz), 2.05–2.02 (m, 2H). ^{13}C NMR ($CDCl_3$, 75 MHz) δ: 172.7, 145.6, 128.7, 121.0, 66.0, 54.7, 40.7, 35.7, 35.0, 22.8.

***N,N,N*-trimethyl-4-(phenylpropyl)amino-4-oxobutan-1-aminium iodide 4t**

Yellow oil. Yield: 74%. MS (ESI) *m/z*: 293.4 (M + H)$^+$. ^{1}H NMR ($CDCl_3$, 300 MHz) δ: 7.24 (q, 5H, *J*= 5.9 Hz), 6.96 (bs, 1H), 3.71 (s, 2H), 3.33–3.25 (m, 9H), 2.66 (t, 2H, *J*= 6.9 Hz), 2.46 (s, 2H), 2.12 (s, 2H), 1.87–1.83 (m, 4H).^{13}C NMR ($CDCl_3$, 75 MHz) δ: 172.7, 138.1, 128.9, 128.2, 126.1, 66.0, 54.9, 40.0, 35.0, 33.1, 29.1, 22.8.4

As reported in figure 1c, γ-amino butyric acid **1** has subjected to Eschweiler-Clarke reductive amination reaction with formaldehyde and formic acid [1] giving 4-(dimethylamino)butanoic acid **2.** It has treated with suitable amines in presence of DCC, HOBt and trimethylamine, in THF or CH_2Cl_2, at room temperature for 24 h (N. Kanamitsu et al.,2007). The amides **3a–t** have been obtained. For compounds **3a–g** a simple extraction has been sufficient to give moderate-good yields of pure products, instead to obtain pure amides **3 h-j, 3 l, 3 o, 3 p, 3 q, 3s** and **3t,** purification on silica gel, by a mixture of $CHCl_3$/MeOH/Et_3N (90:10:0,5), has been necessary.

All compounds **3a-t** were fully characterized by spectroscopic analysis (^{1}HNMR, ^{13}CNMR and mass spectra) after purification.

It was not possible to isolate the amide **3k** with above described procedure, due to the low reactivity of the aromatic amine (4-nitroaniline). Thus, a new procedure was followed, by reacting the 4-nitroaniline with phosphorous trichloride, in pyridine at 40 °C [2]. Finally, the desidered ammonium salts **4a–t** were obtained by simple methylation reaction with iodomethane in acetone for 18 h [3]. After crystallization with diethyl ether, they have been obtained in good yield (64–98%).

The amines used in reductive amination reaction contained short and long chains, cyclic aliphatic, aromatic and phenyl alkyl groups (Supplementary Table S1). The aromatic amines were chosen considering the spacer between the amino group and the aromatic ring (from none spacer to a spacer up to three carbon atoms) and taking into account of the aromatic substituents nature in terms of electron withdrawing or electrondonor groups. Supplementary Table S2 reported the obtained ammonium salts **4a–t.**

Table S1. Amines used for the preparation of **4a–t**.

Compound	R-	Compound	R-
**4a**	$-NHCH_2CH_2CH_3$	**4k**	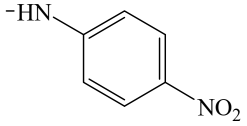
**4b**	$-NHCH_2CH_2CH_2CH_3$	**4l**	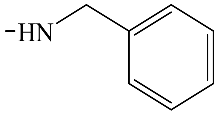
**4c**	$-NHCH_2CCH$	**4m**	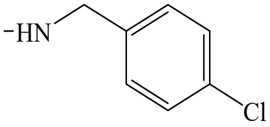
**4d**	$-NHCH_2CH_2CH_2OCH_3$	**4n**	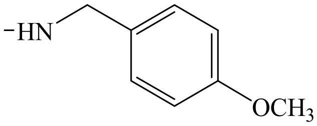
**4e**	$-NHCH_2(CH_2)_{14}CH_3$	**4o**	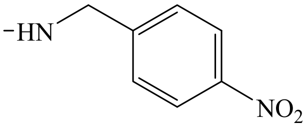
**4f**	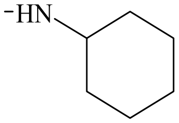	**4p**	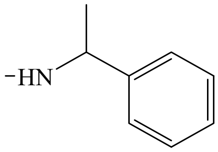
**4g**	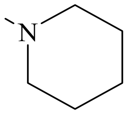	**4q**	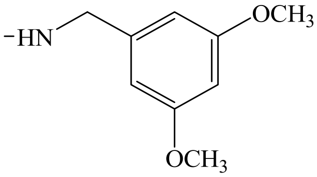
**4h**	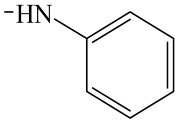	**4r**	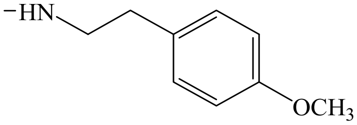
**4i**	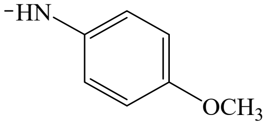	**4s**	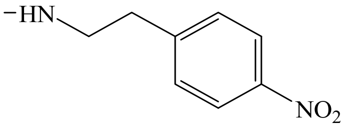
**4j**	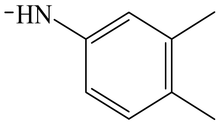	**4t**	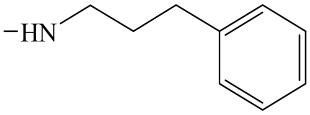

Table S2. Ammonium salts **4a–t**.

Compound	Structure	Compound	Structure
**4a**	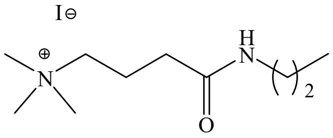	**4b**	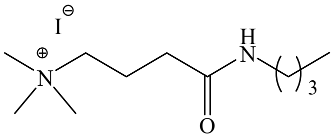
**4c**	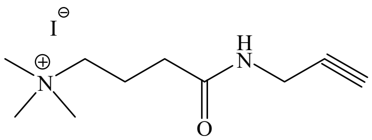	**4d**	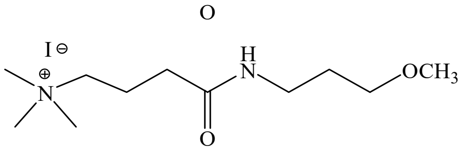
**4e**	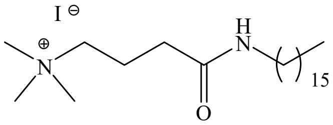	**4f**	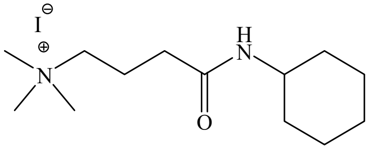
**4g**	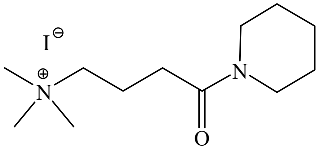	**4h**	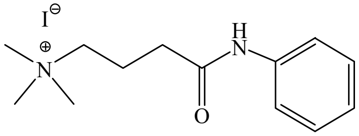
**4i**	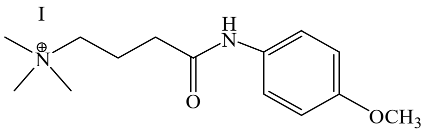	**4j**	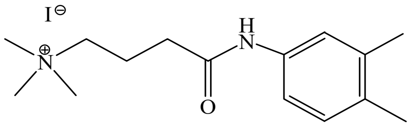
**4k**	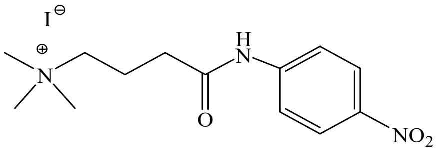	**4l**	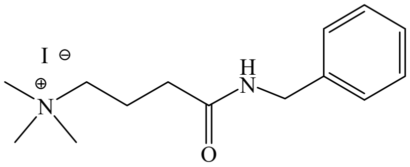

**4m**	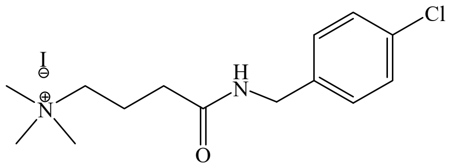	**4n**	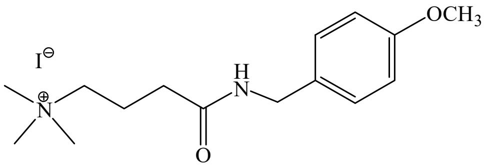
**4o**	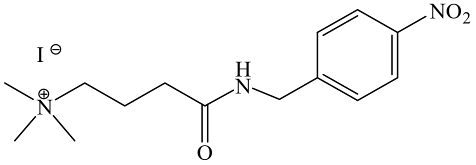	**4p**	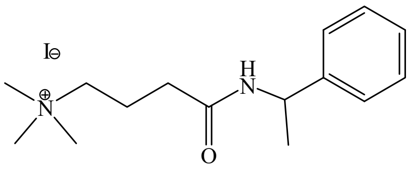
**4q**	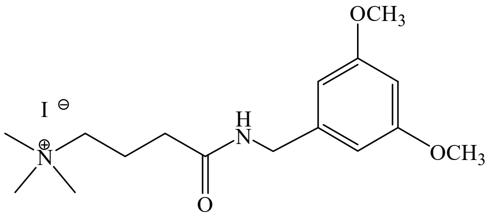	**4r**	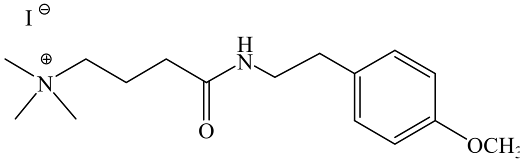
**4s**	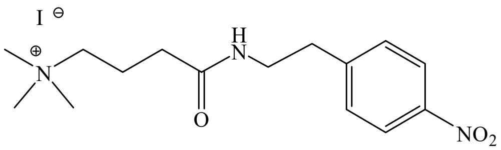	**4t**	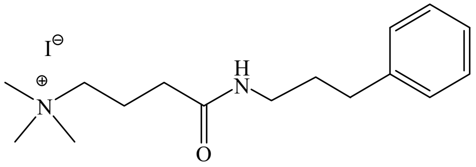

Table S3. The different hOCTN2 protein residues involved in ligand binding.

Compound	Binding Constant (Ki)	Residues Involved in Polar Interactions (Distances in Å)	Residues Involved into Hydrophobic Interactions
**4i**	35 μM	Gln 207 (3.2)<br>Tyr 211 (2.6)<br>Trp 351 (3.2)	Phe 41, Phe 149, Tyr 211, Tyr 239, Trp 351, Ile 354, Phe 443, Val 446, Tyr 447
**4j**	10.8 μM	Tyr 211 (3.0)<br>Ser 470 (2.9)	Ile 208, Tyr 211, Tyr 239, Trp 351, Ile 354, Val 446, Tyr 447
**4k**	8.6 μM	Asn 32 (3.0)<br>Gln 207 (2.6)<br>Tyr 211 (2.5)	Phe 149, Tyr 211, Cys236, Tyr 239, Trp 351, Ile 354, Phe 443, Val 446, Tyr 447
**4t**	10.5 μM	Tyr 211 (2.9)	Phe 41, Phe 149, Val 153, Ile 208, Tyr 211, Tyr 239, Trp 351, Ile 354, Phe 443, Val 446, Tyr 447
**THP**	1.7 mM	Tyr 211 (2.7)<br>Tyr 239 (3.0)<br>Trp 351 (3.0)	Tyr 211, Tyr 239, Trp 354, Tyr 447

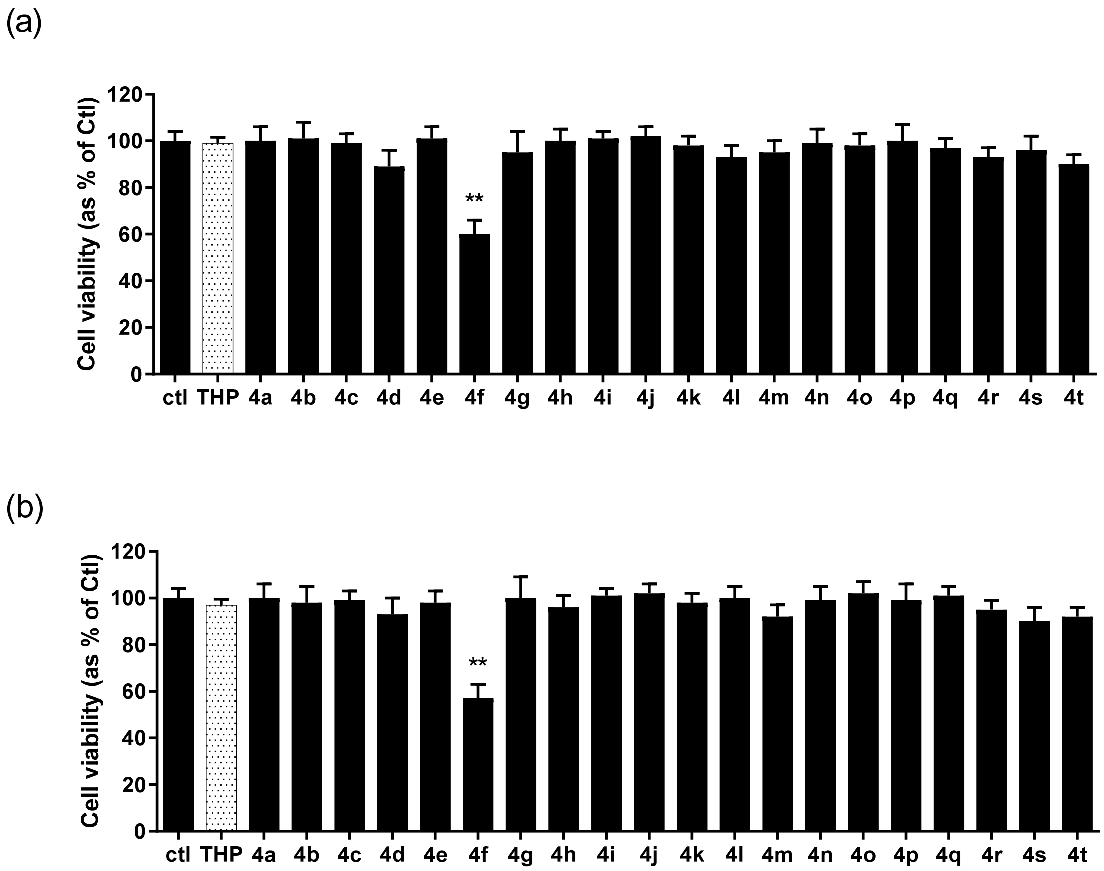

Figure S1. Cell viability of (**a**) STHdh$^{Q7/7}$ and (**b**) STHdh$^{Q111/111}$ cell lines incubated for 72 h in presence of 50 μM of compounds. Bars represent the mean cell viability percentage normalised to untreated control (mean ± SD; n = 3). Statistical significance: *** p< 0.001 versus CTL.

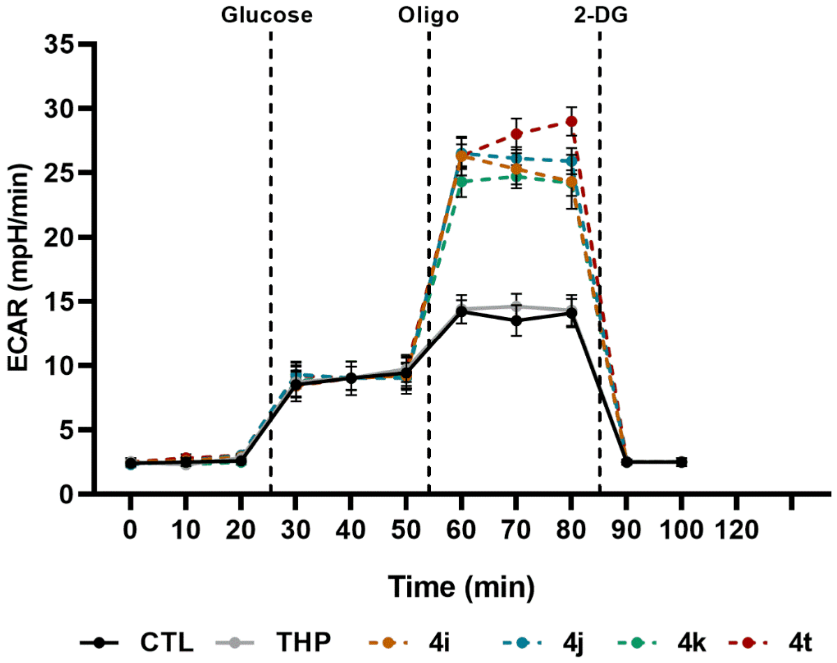

Figure S2. Kinetic profile of ECAR in STHdhQ111/111 cells treated with THP or selected TCL. Data are expressed as mean ± S.E.M. of three independent experiments, each of them in triplicate. ECAR was measured in real time, under basal conditions and in response to glucose, oligomycin, and 2-DG.

References

1. T.C. Bruice, S.J. Benkovic, A Comparison of the Bimolecular and Intramolecular Nucleophilic Catalysis of the Hydrolysis of Substituted Phenyl Acylates by the Dimethylamino Group, *J. Am. Chem. Soc.* **1963**, *85*, 1–8.
2. L. Guo, D. Wang, P.J. Edwards, N. Shobana, R.W. Roeske, Studies on synthesis of peptides with C-terminal glutamine paranitroanilide. In *Peptides Frontiers of Peptide Science*, Springer **2002**, pp. 297–298.
3. Tars, K.; Leitans, J.; Kazaks, A.; Zelencova, D.; Liepinsh, E.; Kuka, J.; Makrecka, M.; Lola, D.; Andrianovs, V.; Gustina, D.; et al. Targeting carnitine biosynthesis: discovery of new inhibitors against gamma-butyrobetaine hydroxylase. *J. Med. Chem.* **2014**, *57*, 2213–2236.
